# Genetic Variants Affect Distinct Metabolic Pathways in Pediatric Multisystem Inflammatory Syndrome and Severe COVID‐19

**DOI:** 10.1002/jmv.70556

**Published:** 2025-08-16

**Authors:** Alysson Henrique Urbanski, Flávia Cristina de Paula Freitas, Tiago Minuzzi Freire da Fontoura Gomes, Michelle Orane Schemberger, Bárbara Carvalho Santos dos Reis, Flavia Amêndola Anísio de Carvalho, Roberta Soares Faccion, Lucas de Almeida Machado, Deborah Antunes dos Santos, Daniela Prado Cunha, Margarida dos Santos Salú, Daniella Campelo Batalha Cox Moore, Mayra Marinho Presibella, Juliana Fontes Noguchi, Henrique Lira Borges, Lais Kimie Tomiura, Luiza Silva de Castro, Letícia Graziela Costa Santos, Esdras Matheus Gomes da Silva, Vinícius Da Silva Coutinho Parreira, Luis Gustavo Morello, Fabricio Klerynton Marchini, Maria Regina Tizzot, Mauricio Marcondes Ribas, Gilberto Pascolat, Carmen Australia Paredes Marcondes Ribas, Fábio Fernandes da Rocha Vicente, Alexandre Rossi Paschoal, Rubens Cat, Benilton de Sá Carvalho, Jaqueline Carvalho de Oliveira, Marcus F. Oliveira, Luiz Lehmann Coutinho, Acácia Maria Lourenço Francisco Nasr, Irina Nastassja Riediger, Jeanine Marie Nardin, Liya Regina Mikami, Ana Carolina Ramos Guimarães, Patricia Savio de Araujo‐Souza, Arnaldo Prata‐Barbosa, Zilton Farias Meira de Vasconcelos, Helisson Faoro, Hellen Geremias dos Santos, Fabio Passetti

**Affiliations:** ^1^ Instituto Carlos Chagas FIOCRUZ Curitiba Paraná Brazil; ^2^ Universidade Federal de São Carlos São Carlos São Paulo Brazil; ^3^ Departamento de Bioquímica e Biologia Tecidual, Instituto de Biologia Universidade Estadual de Campinas Campinas São Paulo Brazil; ^4^ Laboratório de Alta Complexidade (LACIFF), Unidade de Pesquisa Clínica, Instituto Fernandes Figueira FIOCRUZ Rio de Janeiro Brazil; ^5^ Laboratório de Genômica Aplicada e Bioinovações (IOC), Instituto Oswaldo Cruz FIOCRUZ Rio de Janeiro Brazil; ^6^ Programa de Pós‐graduação em Pesquisa Aplicada à Saúde da Criança e da Mulher, Instituto Nacional de Saúde da Mulher da Criança e do Adolescente Fernandes Figueira (IFF), FIOCRUZ Rio de Janeiro Brazil; ^7^ Pediatric Intensive Care Unit Hospital Martagão Gesteira Salvador Bahia Brazil; ^8^ Departamento de Pediatria, IFF Unidade de Pacientes Graves FIOCRUZ Rio de Janeiro RJ Brazil; ^9^ Faculdade de Medicina Universidade Federal Fluminense Niterói Rio de Janeiro RJ Brazil; ^10^ Laboratório Central do Estado do Paraná (LACEN) São José dos Pinhais Paraná Brazil; ^11^ Faculdade Evangélica Mackenzie Do Paraná Curitiba Paraná Brazil; ^12^ Hospital Universitário Evangélico Mackenzie Curitiba Paraná Brazil; ^13^ Federal University of Technology ‐ Paraná (UTFPR) Cornélio Procópio Paraná Brazil; ^14^ Departamento de Pediatria Universidade Federal do Paraná Curitiba Paraná Brazil; ^15^ Departamento de Estatística Universidade Estadual de Campinas Campinas São Paulo Brazil; ^16^ Departamento de Genética Universidade Federal do Paraná Curitiba Paraná Brazil; ^17^ Universidade Federal do Rio de Janeiro Rio de Janeiro Brazil; ^18^ Instituto Nacional de Ciência e Tecnologia em Entomologia Molecular Rio de Janeiro Rio de Janeiro Brazil; ^19^ Escola Superior de Agricultura Luiz de Queiroz (ESALQ) Universidade de São Paulo Piracicaba São Paulo Brazil; ^20^ Secretaria da Saúde do Estado do Paraná (SESA) Curitiba Paraná Brazil; ^21^ Escola de Medicina e Ciências da Vida (EMCV) Pontifícia Universidade Católica do Paraná – PUCPR Curitiba Paraná Brazil; ^22^ Universidade Federal do Paraná Curitiba Paraná Brazil; ^23^ Instituto D′or de Pesquisa e Ensino Rio de Janeiro Brazil

**Keywords:** carbohydrate metabolism, cholesterol metabolism, COVID‐19, genetic variants, MIS‐C (multisystem inflammatory syndrome in children), whole‐exome sequencing

## Abstract

The coronavirus disease 2019 (COVID‐19) pandemic has triggered a global health crisis, with over 700 million confirmed cases and at least 7 million deaths reported by early 2024. Children are less vulnerable to severe SARS‐CoV‐2 infection than adults and typically experience milder respiratory symptoms. However, a rare but significant complication, known as multisystem inflammatory syndrome in children (MIS‐C), can develop weeks after infection, characterized by a spectrum of inflammatory symptoms. This study employed whole‐exome sequencing and over‐representation analysis to identify genetic variants of potential clinical significance related to MIS‐C or severe COVID‐19 in a group of children with acute respiratory distress syndrome (ARDS), all of whom were unvaccinated for COVID‐19. We observed the enrichment of potentially pathogenic genetic variants in genes related to carbohydrate metabolism, particularly glycogen breakdown, in severe COVID‐19 pediatric patients, and in genes related to cholesterol and lipoprotein metabolism in MIS‐C patients. These findings offer insights into the genetic underpinnings of MIS‐C and severe COVID‐19, suggesting potential genes and biological pathways for further research.

## 1 Introduction

The coronavirus disease 2019 (COVID‐19) pandemic, caused by the severe acute respiratory syndrome coronavirus 2 (SARS‐CoV‐2), has resulted in a major global health crisis. By early 2025, over 700 million confirmed cases and more than seven million deaths had been reported [1]. Clinical manifestations range from asymptomatic or mild upper respiratory tract symptoms to severe pneumonia and acute respiratory distress syndrome (ARDS), which can lead to respiratory failure and death [2]. Pre‐existing conditions – including overweight, diabetes, cardiac disease, age over 60 years – significantly increase the risk of severe disease and mortality [3].

In children, COVID‐19 typically manifests with milder symptoms than in adults [4]. However, a rare Kawasaki‐like presentation known as Multisystem Inflammatory Syndrome in Children (MIS‐C) has emerged [5], characterized by fever, rash, shock, gastrointestinal and neurological symptoms, myocarditis, and coagulopathy [6]. MIS‐C usually develops three to 5 weeks following a SARS‐CoV‐2 infection [7].

The genetic mechanisms underlying pediatric COVID‐19 ARDS and MIS‐C remain unclear. Evidence suggests genetic variants in cytokine‐related genes [8], ACE2 [9], and inborn errors of immunity [10] may predispose to severe disease. Other studies have further implicated genes related to immune and inflammatory disorders in MIS‐C pathogenesis [11, 12, 13, 14, 15].

The present study provides a comprehensive analysis of potentially pathogenic exonic variants with low or unknown population frequency in public databases, identified in unvaccinated pediatric patients who experienced severe COVID‐19 (sCOVID‐19) or MIS‐C. The primary objective was to determine which genes and pathways may be involved in these severe pediatric manifestations, thereby offering insights into the pathogenesis of SARS‐CoV‐2 infection in children. The findings highlight genetic variants that may confer susceptibility to severe outcomes in pediatric patients, informing genetic screening approaches and therapeutic strategies.

## 2 Methods

### 2.1 Subjects and Definition of Phenotypes

This prospective, multicenter cohort study (September 2020–August 31, 2021) was conducted in private and public institutions across the Brazilian states of Bahia, Rio de Janeiro, São Paulo and Paraná. The participating institutions included Instituto Fernandes Figueira (Rio de Janeiro), Hospital Real D′Or (Rio de Janeiro), Hospital Alvorada (São Paulo), Hospital Universitário Pedro Ernesto (Rio de Janeiro), Unimed Leste Fluminense (São Gonçalo), Hospital Martagão Gesteira (Salvador), Complexo Hospitalar de Niterói (Niterói), Hospital Quinta D′Or (Rio de Janeiro), and Hospital Universitário Evangélico Mackenzie (Curitiba).

Data from the MIS‐C cohort study by Reis et al. (2023) were included in this study as part of a project approved by the Internal Review Board (IRB) of the Instituto D′Or de Pesquisa e Ensino (IDOR), the proponent institution, under CAAE no. 30272920.0.1001.5249. Each participating institution also received approval from its respective IRB. The remaining COVID‐19 and MIS‐C samples were part of projects approved by the Human Research Ethics Committee of the Faculdade Evangélica Mackenzie do Paraná under CAAE no. 55543322.2.0000.0103 and by the Human Research Ethics Committee of the Health Sciences Sector of the Federal University of Paraná under CAAE no. 55543322.2.3001.0102.

**Figure 1:**
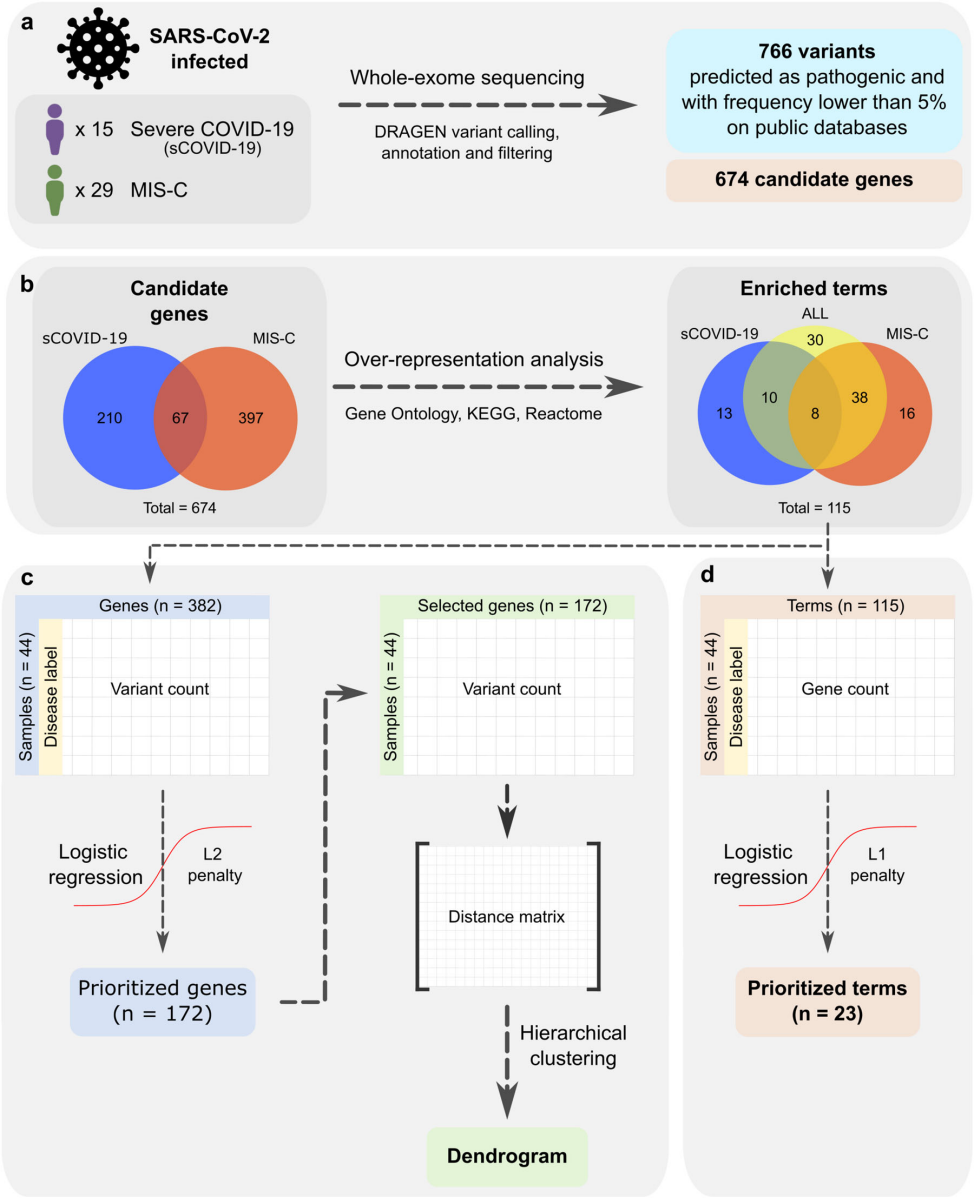
Study design overview. (a) Whole‐exome sequencing was performed on 44 pediatric patients infected with SARS‐CoV‐2 – 15 diagnosed with severe COVID‐19 (sCOVID‐19) and 29 with Multisystem Inflammatory Syndrome in Children (MIS‐C) – yielding 766 filtered variants across 674 candidate genes. (b) Over‐representation analysis (ORA) using Gene Ontology (GO), Kyoto Encyclopedia of Genes and Genomes (KEGG), and Reactome databases was conducted on sCOVID‐19‐specific, MIS‐C‐specific, and shared candidate genes, resulting in 115 enriched biological terms (*q* < 0.01 for GO/KEGG, *q* < 0.02 for Reactome). (c) From 382 ORA‐enriched genes, 172 were prioritized using logistic regression with an L2 penalty (coefficient ≥ |0.13 |). Hierarchical clustering based on Euclidean distance was then applied to these prioritized genes to group patient samples. (d) Of the 115 enriched terms identified, 23 were found to be most associated with sCOVID‐19 or MIS‐C after applying logistic regression with an L1 penalty, highlighting key pathways that distinguish the two conditions.

Two groups of patients were defined based on symptom progression: severe COVID‐19 (sCOVID‐19) and MIS‐C, all of whom were unvaccinated for COVID‐19 at the time of sample collection. sCOVID‐19 was defined according to acute respiratory distress syndrome criteria: hypoxemic respiratory failure, hypoxemia, and need for mechanical ventilation. MIS‐C was defined based on the World Health Organization criteria for patients aged 0‐19 years with persistent fever ( ≥ 3 days) along with least two of the following: (1) Rash or bilateral non‐purulent conjunctivitis or muco‐cutaneous inflammation signs (oral, hands, or feet); (2) Hypotension or shock; (3) Myocardial dysfunction, pericarditis, valvulitis, or coronary abnormalities (by ECHO or elevated Troponin/NT‐proBNP); (4) Coagulopathy (abnormal PT/PTT, elevated d‐Dimers); (5) Acute gastrointestinal symptoms (diarrhea, vomiting or abdominal pain); (6) Elevated inflammatory markers (ESR, C‐reactive protein or procalcitonin) and no other obvious microbial cause of inflammation, including bacterial sepsis; (7) Evidence of infection by SARS‐CoV‐2 (RT‐PCR, antigen test or positive serology) or likely contact with infected patients. The sCOVID‐19 group included 15 pediatric patients, while the MIS‐C group comprised 29 (Figure 1a).

### 2.2 DNA Extraction, Whole‐Exome Sequencing, and Variant Calling

Peripheral blood DNA was extracted from 29 MIS‐C and 10 sCOVID‐19 samples using the QIAamp DNA Blood Mini Kit (QIAGEN). Library preparation was performed with the Illumina Exome Panel, followed by paired‐end sequencing (2×100 bp) on a NextSeq. 2000 at Centro de Genômica Funcional (ESALQ/USP, Piracicaba, Brazil). An additional five sCOVID‐19 samples underwent DNA extraction from buccal mucosa using the QIAamp 96 DNA QIAcube HT kit (QIAGEN). Libraries were prepared with the Illumina DNA Prep with Exome 2.0 Enrichment kit and sequenced (2×100 bp) on an Illumina NovaSeq. 6000 RPT01P/Carlos Chagas ‐ Fiocruz Paraná.

Adapter trimming and quality filtering were performed using Trimmomatic [16], and FASTQ files were mapped to the GRCh37 reference genome with the Illumina DRAGEN Bio‐IT Platform (v4.2), retaining only primary alignments. All samples were verified to have a > 45× mean coverage. Variant calling was conducted using the DRAGEN Small Variant Caller with machine learning‐based recalibration enabled.

### 2.3 Kinship Inference

To exclude hidden familial relationships, we estimated pair‐wise kinship coefficients with KING (v2.2.7) [17]. Autosomal, biallelic SNPs were extracted from the joint multi‐sample VCF and filtered as follows: quality flag = PASS; minor‐allele frequency ≥ 5%; genotype missingness ≤ 5%. Linkage‐disequilibrium pruning was performed in PLINK (v2.0) [18] (‐‐indep‐pairwise 50 5 0.2), and the pruned data set was converted to binary PLINK format for KING analysis with default parameters. All kinship coefficients (Φ) were below the 0.044 threshold for third‐degree relatives, confirming that every participant can be treated as unrelated in downstream analyses.

### 2.4 Variant Annotation and Filtering

Variants were annotated using Nirvana (v3.18) [19], including clinical‐grade and populational frequency annotations. Variables from Nirvana included variant consequence from VEP (v91), RefSNP (dbSNP 155), REVEL (v20200205), phyloP (v20091110), GERP++ (v20110522), ClinVar (v20230822), allele frequencies from the Greater Middle East Variome (v20160618), and from all ancestry populations derived from 1000 genomes Phase 3 v5a (African, Admixed American, East Asian, European, and South Asian) and gnomAD v2.1 (African, Latino/Admixed American, Ashkenazi Jewish, East Asian, Finnish, Non‐Finnish European, South Asian, and Other). Brazilian population allele frequencies were subsequently incorporated from the ABraOM (vSABE‐609‐WES) and DNABR databases [20, 21].

The final list of variants was filtered based on variant consequence according to Sequence Ontology terms [22], including missense variants, in frame insertions and deletions, stop‐gained, start‐lost, frameshift variants, splice region variants, splice site variants, and nonsense‐mediated mRNA decay. Only variants with REVEL scores ≥ 0.773 [23] or classified as pathogenic in at least one ClinVar submission were retained. Additionally, only variants with a frequency < 5% (or lacking frequency data) across all populations were selected, and a subset analysis used a < 1% cutoff. Variants in the *MADCAM1* gene were removed due to a high frequency bias in our samples, as they were no longer classified as low‐frequency following the gnomAD v4 update. Variants were additionally annotated with single‐nucleotide conservation scores: PhyloP 46‐way and GERP++ RS. These metrics were employed solely for descriptive stratification and never as inclusion or exclusion filters. On the basis of established cut‐offs that mark strong purifying selection [24, 25], each variant was placed in one of five categories: (i) Highly conserved (both PhyloP > 2 and GERP RS > 4); (ii) Conserved (PhyloP) (PhyloP > 2 but GERP RS ≤ 4); (iii) Conserved (GERP) (GERP RS > 4 but PhyloP ≤ 2); (iv) Low conservation (neither threshold satisfied); (v) Unscored (both scores missing).

Variant pathogenicity was then appraised with the Franklin by QIAGEN platform, which implements the 2015 American College of Medical Genetics and Genomics/Association for Molecular Pathology (ACMG/AMP) framework [26, 27]. Franklin automatically assigns evidence codes (PS, PM, PP, BS, BP) and one of five standard categories: pathogenic, likely pathogenic, variant of uncertain significance, likely benign, or benign. As with the conservation scores, ACMG/AMP classifications informed downstream interpretation only and were not employed as inclusion or exclusion criteria during variant selection.

### 2.5 Over‐Representation Analysis

Over‐Representation Analysis (ORA) was applied to identify over‐represented terms among genes carrying potentially pathogenic variants. ORA was performed using Gene Ontology (GO), Kyoto Encyclopedia of Genes and Genomes (KEGG), and Reactome databases via R packages (clusterProfiler v4.10, DOSE v3.28.2, GOSemSim v2.28, and ReactomePA v1.46.0) [28, 29, 30, 31]. For each group (sCOVID‐19, MIS‐C, and all samples), the input included all genes with identified variants (not limited to exclusive genes). Enriched terms were retained if they had q‐values < 0.01 (GO/KEGG) or < 0.02 (Reactome).

### 2.6 Gene and Term Selection

To reduce dimensionality for hierarchical clustering, genes were prioritized using logistic regression with an L2 penalty (Ridge regression) in Python′s scikit‐learn [32]. The model used disease status (sCOVID‐19 or MIS‐C) as the response variable and included 382 covariates, representing gene variant counts from ORA‐enriched terms with q‐values < 0.01 (GO/KEGG) or < 0.02 (Reactome). Genes with regression coefficients ≥ |0.13| were retained, yielding 172 genes for hierarchical clustering.

The most relevant enriched terms were identified using logistic regression with an L1 penalty (Least Absolute Shrinkage and Selection Operator ‐ LASSO), with disease status as the response and 115 enriched terms as covariates, using default scikit‐learn parameters. These terms, each representing the number of genes with variants per patient, were selected based on ORA results with q‐values < 0.01 (GO/KEGG) or < 0.02 (Reactome).

### 2.7 Hierarchical Clustering

Hierarchical clustering was performed using per‐patient variant counts for the 172 genes with regression coefficients ≥ |0.13| from the L2 logistic regression model. Euclidean distances between patients were calculated using Python′s scipy [33], and clustering was executed using Ward′s method. The final number of clusters was determined by visual inspection of the dendrogram and assessment of cluster interpretability.

### 2.8 Three‐Dimensional Protein Modeling

Protein sequences, structural features and functional annotations were retrieved from UniProtKB [34] for the four proteins that were taken forward to three‐dimensional modelling: ABCB11 (O95342), CFTR (P13569), GALT (P07902) and PYGM (P11217). These variants – GALT p. Gln188Arg (rs75391579), PYGM p. Ala365Val (rs116135678), CFTR p. Leu206Trp (rs121908752) and ABCB11 p. Arg517His (rs760750012) – were chosen because each met three stringent filters: (i) high predicted pathogenicity (REVEL ≥ 0.80), (ii) an ACMG/AMP classification of pathogenic, and (iii) strong evolutionary constraint (PhyloP > 4.0 and GERP++ RS > 4.5). An expanded protein modeling analysis with ABCA5 (Q8WWZ7), ABCB4 (P21439), ADIPOQ (Q15848), APOE (P02649), AMY2B (P19961), GAA (P10253), GALE (Q14376), GANC (Q8TET4), NR1H3 (Q13133), PHKA1 (P46020), PGM1 (P36871), and SI (P14410) is available in Supplementary Methods.

The 3.2 Å cryo‐EM structure of ABCB11 (PDB ID 8PMD), the 2.80 Å cryo‐EM structure of CFTR (PDB ID 8EIO), the 1.73 Å crystal structure of GALT (PDB ID 5IN3), and the 2.30 Å crystal structure of PYGM in complex with AMP and glucose (PDB ID 1Z8D) were obtained from the Protein Data Bank (PDB).

For the trans‐membrane transporters, the positioning and tilt angle relative to the lipid bilayer were estimated using the Positioning of Proteins in Membrane (PPM) server [35], which applies an empirically adjusted, physically based implicit membrane model. Missense variants were modeled into the protein structures using the UCSF ChimeraX [36] “swapaa” command guided by Dunbrack backbone‐dependent rotamer libraries [37], and hydrogen bond interactions along with three‐dimensional structural representations were analyzed using ChimeraX′s default settings.

## 3 Results

### 3.1 Whole‐Exome Sequencing and Variant Calling

**Figure 2:**
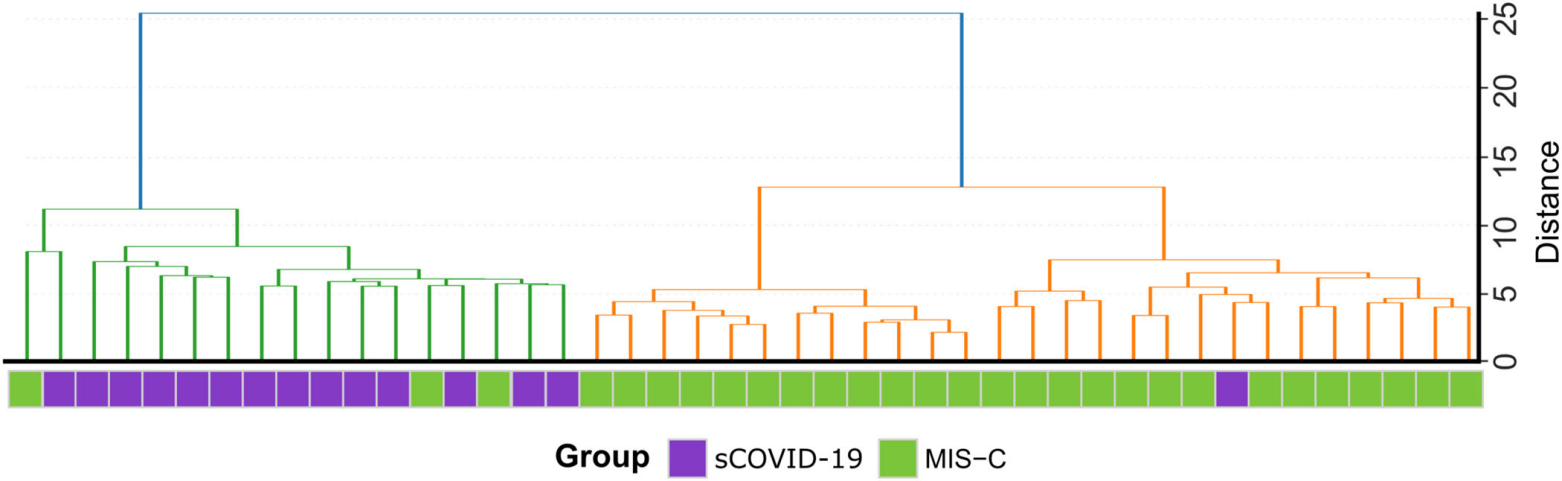
Hierarchical clustering of patient samples. Hierarchical clustering was conducted using the per‐gene variant counts for each of the 44 pediatric patients (sCOVID‐19 in purple, MIS‐C in green). Euclidean distances were computed and Ward′s method was applied, revealing two principal clusters: Cluster 1 (green) predominantly includes sCOVID‐19 samples, whereas Cluster 2 (orange) is mainly composed of MIS‐C cases. This clustering indicates distinct genetic variant profiles between the two clinical groups.

Whole‐exome sequencing (WES) was conducted on 15 pediatric patients with severe COVID‐19 (sCOVID‐19) (8 females [53.3%], 7 males [46.7%]; median age: 8 months [range: 1–180]) and 29 with MIS‐C (18 females [62.1%], 11 males [37.9%]; median age: 72 months [range: 2–192]) (Wilcoxon test, *p* = 0.019; Supporting Information S1: Figure 1). The mean [SD] WES coverage across all samples was 67.5× [26.9], including 80.85× [38.7] for sCOVID‐19 samples and 60.54× [14.6] for MIS‐C samples (Supporting Information S1: Figure 2). Kinship analysis based on autosomal SNPs returned Φ < 0.044 for all 946 pairwise comparisons, indicating that none of the 44 individuals are related at the third‐degree level or closer.

Variant calling identified 203 815 genetic variants. After applying filters for variant consequence, predicted pathogenicity, and population frequency (< 5% or unreported), 766 variants remained (Figure 1a). Among these, 730 were single nucleotide variants, 23 were deletions, and 13 were insertions. Specifically, variants were retained if they were predicted to be potentially pathogenic by REVEL (*n* = 596), had at least one pathogenic entry in ClinVar (*n* = 197), or exhibited unknown frequency (*n* = 86) or frequencies below 5% (*n* = 680) in ABraOM, gnomAD, and 1000 Genomes. Collectively, these variants affected 674 genes, with 277 identified in sCOVID‐19 and 464 in MIS‐C (Figure 1b).

### 3.2 Over‐Representation Analysis

Over‐Representation Analysis (ORA) identified both shared and distinct biological pathways linked to genetic variants in sCOVID‐19 and MIS‐C. In total, 674 genes carrying these variants (277 in sCOVID‐19 and 464 in MIS‐C) were used as ORA input with GO, KEGG, and Reactome databases. From this analysis, 115 enriched biological terms were identified, comprising 62 specific to MIS‐C, 31 specific to sCOVID‐19, and 8 shared between the two groups (Figure 1b).

### 3.3 Sample Clustering

Sample clustering (Figure 1c) revealed distinct genetic profiles for patients with sCOVID‐19 and MIS‐C. Hierarchical clustering was performed using Euclidean distances on the variant counts from 172 genes selected through logistic regression (L2 penalty, coefficient ≥ |0.13 | ). This approach separated the two clinical groups; however, four samples diverged from the overall pattern (three MIS‐C samples clustered with sCOVID‐19, and one sCOVID‐19 sample clustered with MIS‐C) (Figure 2). These findings suggest distinct patterns of genetic variants in sCOVID‐19 and MIS‐C patients, indicating that different gene sets contribute to the severity and clinical presentation of these conditions.

### 3.4 Distinct Enriched Terms in sCOVID‐19 and MIS‐C Revealed by ORA and Logistic Regression

**Figure 3:**
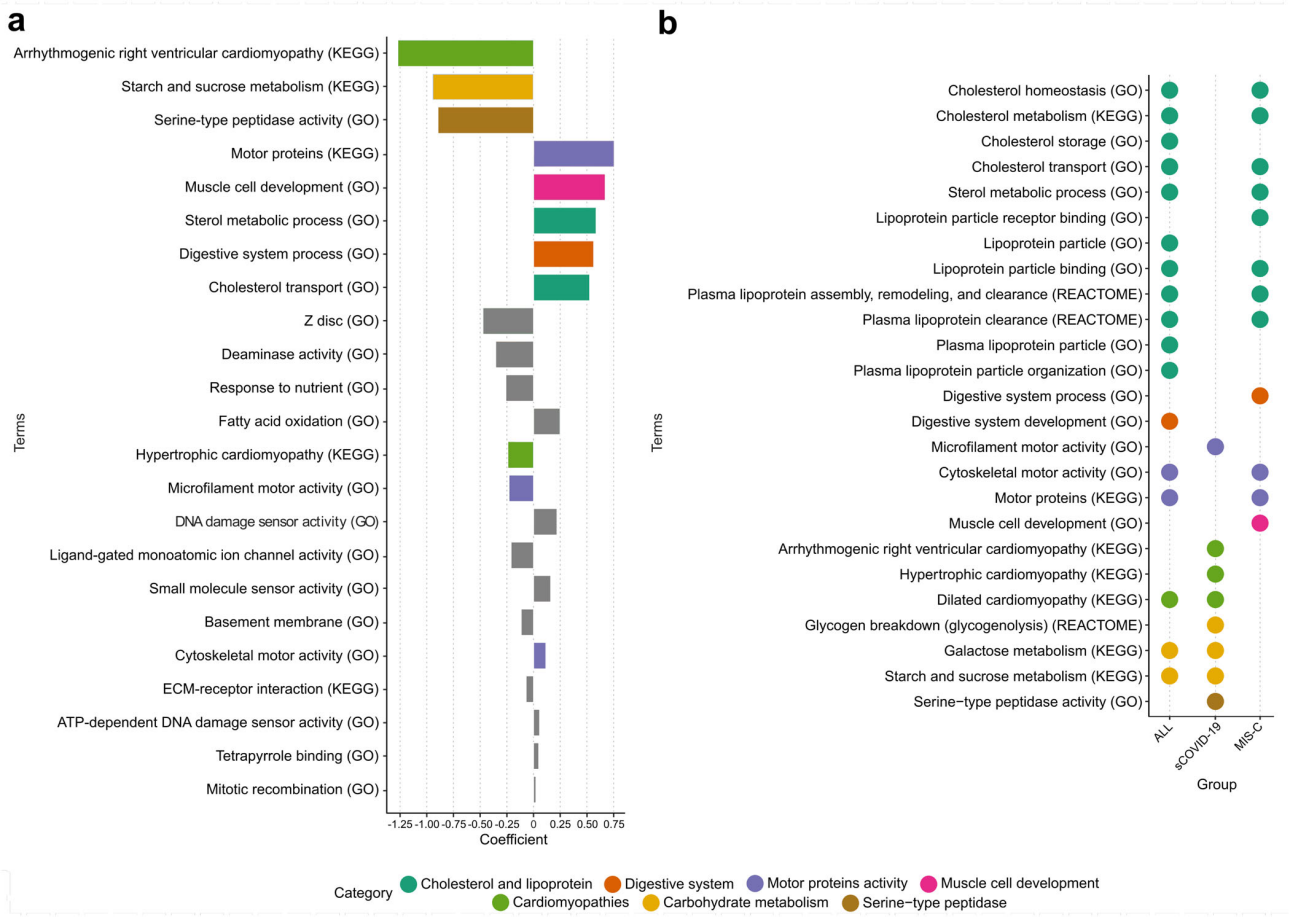
Terms prioritized by logistic regression and over‐representation analysis. (a) Logistic regression with an L1 penalty was applied to 115 enriched terms to identify those most closely associated with either severe COVID‐19 (sCOVID‐19) or MIS‐C. Negative regression coefficients indicate terms more likely linked to sCOVID‐19, whereas positive coefficients indicate those preferentially associated with MIS‐C. (b) Dot plot illustrating the key terms (coefficient > |0.5| in panel a) and closely related enriched terms from the over‐representation analysis (ORA). Terms were derived from GO, KEGG, and Reactome databases, each meeting the respective significance thresholds (*q* < 0.01 for GO/KEGG, *q* < 0.02 for Reactome). Color coding denotes broad functional categories, including cholesterol and lipoprotein metabolism, digestive system, motor protein activity, muscle cell development, cardiomyopathies, carbohydrate metabolism, and serine‐type peptidase activity.

Logistic regression identified terms most likely associated with sCOVID‐19 or MIS‐C based on ORA findings (Figure 1d). This approach yielded 23 prioritized terms, of which 8 demonstrated coefficients exceeding |0.5| (Figure 3a). These terms represented seven categories: cholesterol/lipoprotein metabolism, digestive system processes, motor proteins activity, muscle cell development, cardiomyopathies, carbohydrate metabolism, and serine‐type peptidase activity. These terms guided deeper exploration of related enriched terms in the ORA results (Figure 3b).

Additionally, ORA identified distinct pathways linked to either sCOVID‐19 or MIS‐C (Figure 3b, Supporting Information S1: Data 1). The sCOVID‐19 group exhibited enrichment in cardiomyopathies, carbohydrate metabolism, and serine‐type peptidase activity, whereas MIS‐C showed enrichment in cholesterol and lipoprotein metabolism, the digestive system, motor proteins activity, and muscle cell development. The present analysis focuses on carbohydrate and cholesterol metabolism, as related pathways remained enriched even under a stricter populational variant frequency threshold (< 1% in public databases; Supporting Information S1: Data 2).

### 3.5 Genes Affected in sCOVID‐19 and MIS‐C Enriched Terms

**Figure 4:**
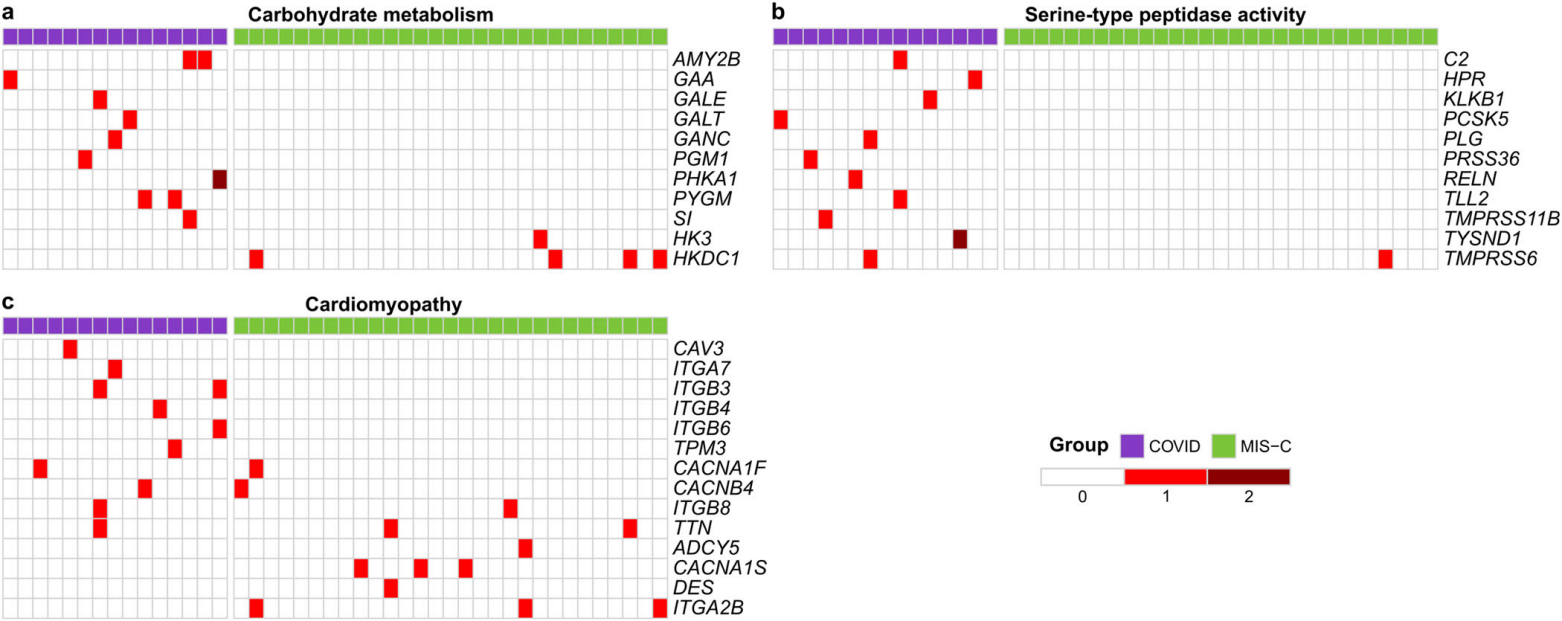
Genes affected in sCOVID‐19‐enriched terms. Heatmaps display the presence of genetic variants in each patient sample (columns) across key genes (rows) within the sCOVID‐19 group (purple) and MIS‐C group (green). The intensity of red indicates the number of variants detected in each gene‐sample pair. Genes are organized into three main categories enriched in sCOVID‐19: (a) Carbohydrate metabolism (Starch and sucrose metabolism [KEGG], Glycogen breakdown [glycogenolysis] [Reactome], and Galactose metabolism [KEGG]). (b) Serine‐type peptidase activity (GO). (c) Cardiomyopathy (Hypertrophic cardiomyopathy [KEGG], Dilated cardiomyopathy [KEGG], and Arrhythmogenic right ventricular cardiomyopathy [KEGG]).

**Figure 5:**
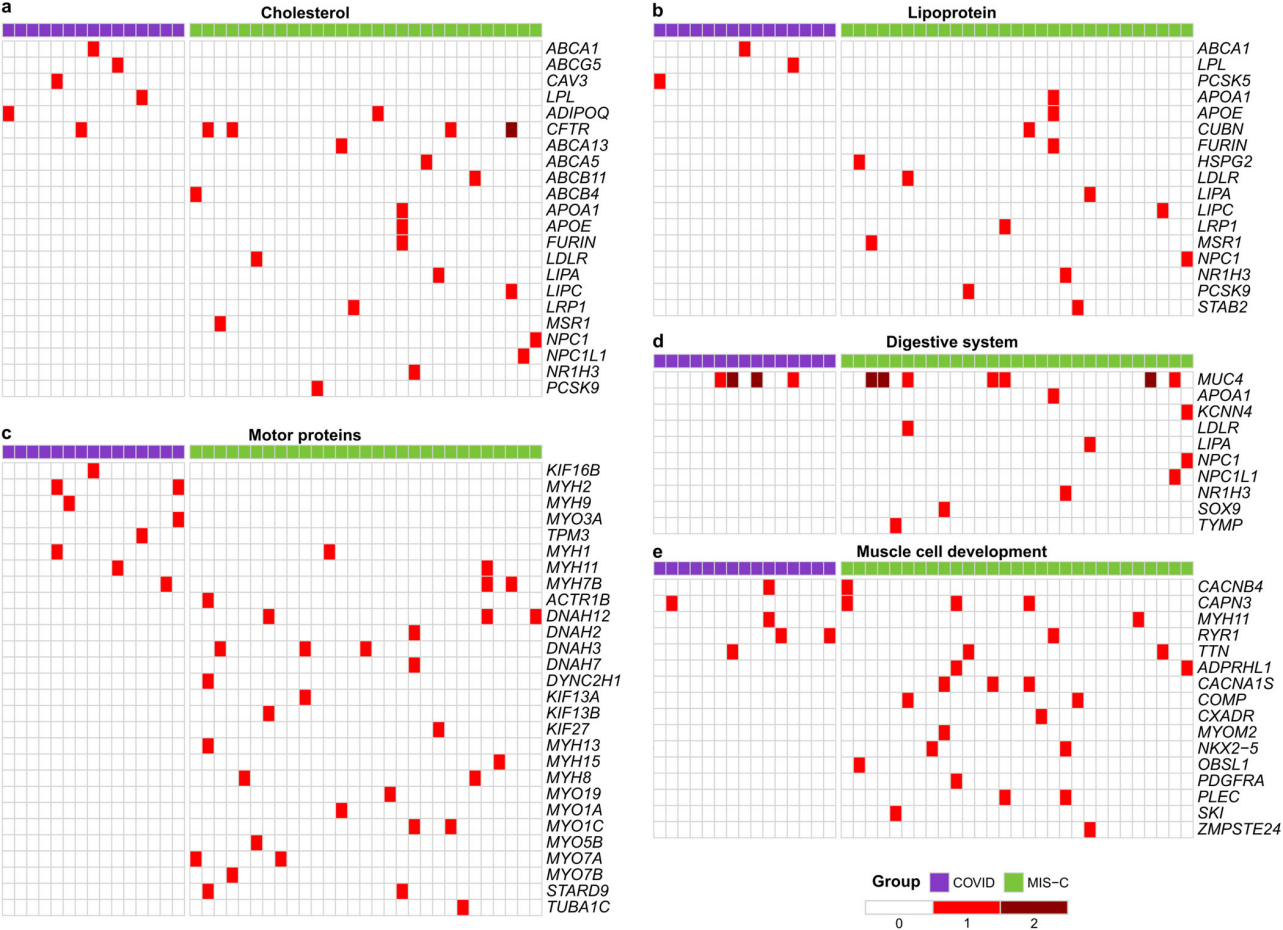
Genes affected in MIS‐C‐enriched terms. Heatmaps display the presence of genetic variants in each patient sample (columns) across key genes (rows) within the sCOVID‐19 group (purple) and MIS‐C group (green). The intensity of red indicates the number of variants detected in each gene‐sample pair. Genes are grouped into categories enriched in MIS‐C. (a) Cholesterol metabolism (Cholesterol transport [GO], Cholesterol metabolism [KEGG], Cholesterol homeostasis [GO]). (b) Lipoprotein metabolism (Plasma lipoprotein clearance [Reactome], Plasma lipoprotein assembly, remodeling, and clearance [Reactome], Lipoprotein particle receptor binding [GO], Lipoprotein particle binding [GO]). (c) Motor proteins (Motor proteins [KEGG], Cytoskeletal motor activity [GO]). (d) Digestive system process (GO). (e) Muscle cell development (GO).

To further explore the pathways enriched in Figure 3b, terms exclusively linked to sCOVID‐19 or MIS‐C were merged, yielding distinct gene lists for each group. Figures 4 and 5 illustrate and quantify the presence of variants in these genes among sCOVID‐19 and MIS‐C samples. The results reinforce the ORA findings and highlight the unique sets of genes that may shape the pathophysiology in each condition.

Among the pathway genes recovered by the over‐representation analysis, an exclusivity filter was applied: genetic variants in carbohydrate‐metabolism genes were retained only when observed in sCOVID‐19 cases, whereas genetic variants in cholesterol/lipoprotein‐metabolism genes were retained only when observed in MIS‐C cases. This pathway–phenotype criterion yielded 34 rare, putatively deleterious genetic variants distributed across 30 genes (Table 1). The resulting set segregates strictly by phenotype: nine carbohydrate‐metabolism genes carry sCOVID‐19‐specific genetic variants, whereas 21 cholesterol/lipoprotein‐metabolism genes carry MIS‐C‐specific genetic variants

**Table 1:** Shortlist of genetic variants in carbohydrate and cholesterol/lipoprotein metabolism genes.^a^

Gene	HGVSG	dbSNP	REVEL	ACMG criteria	ACMG classification	PhyloP	GERP	Conservation classification
ABCA13	NC_000007.13:g.48547481 C > T	rs76060602	0.785	PM2, BS2, PP3, BP6	B	3.6	5.5	Highly conserved
ABCA5	NC_000017.10:g.67251772 T > A	—	0.959	PP3, PM2	LP	4.8	5.62	Highly conserved
ABCB11	NC_000002.11:g.169828445 C > T	rs760750012	0.84	PM3, PS3, PM2, PM1, PP3	P	4.6	5.83	Highly conserved
ABCB4	NC_000007.13:g.87041333 C > T	rs61730509	0.784	PP3, PP2, BA1, BS2	B	5.4	5.48	Highly conserved
ADIPOQ	NC_000003.11:g.186572432 A > G	rs754228738	0.801	PM2, PP3	VUS	2.6	4.91	Highly conserved
AMY2B	NC_000001.10:g.104116544 C > A	rs137860562	0.84	PM2, PP3	VUS	5.5	4.6	Highly conserved
AMY2B	NC_000001.10:g.104117852 G > A	rs147215616	0.929	PM2, BS2, PP3	VUS	4.4	4.35	Highly conserved
APOA1	NC_000011.9:g.116706940_116706942del	rs532489785	—	PM2, PM4, BP6,	VUS	—	—	Unscored
APOE	NC_000019.9:g.45412040 C > T	rs769455	0.695	PM5, PP3, BA1, BS2, PP5, BP6	VUS	0.6	3.98	Low conservation
CFTR	NC_000007.13:g.117175339 T > G	rs121908752	0.903	PM3, PS3, PM1, PP2, PM2, PP3	P	5	5.52	Highly conserved
CFTR	NC_000007.13:g.117176711 A > T	rs151073129	0.917	PP3, PP2, BS1, BP6	B	4.6	5.1	Highly conserved
CFTR	NC_000007.13:g.117267592 G > T	rs1800120	0.944	PP3, PM2, PP2, BP6	LP	5.6	5.77	Highly conserved
CFTR	NC_000007.13:g.117199646_117199648del	rs113993960	—	PS4, PS1, PM4, PM5, PM2	P	—	—	Unscored
CUBN	NC_000010.10:g.17164852 C > T	rs772400701	0.827	PM2, PP3	VUS	5.4	5.22	Highly conserved
FURIN	NC_000015.9:g.91420785 G > T	rs1187222557	0.827	PM2, PP3	VUS	5.4	3.92	Conserved (PhyloP)
GAA	NC_000017.10:g.78078651 G > A	rs200586324	0.819	PP4, PM3, BS3, PM2, PM5, PP3, PP2	LP	5.5	4.94	Highly conserved
GALE	NC_000001.10:g.24124241 C > T	rs374106365	0.931	PM2, PP3, PP2	VUS	5.2	4.72	Highly conserved
GALT	NC_000009.11:g.34648167 A > G	rs75391579	0.975	PM3, PM1, PP2, PM2, PP3, PP5	P	4.3	4.77	Highly conserved
GANC	NC_000015.9:g.42614036 A > T	rs751368632	0.824	PM2, PP3	VUS	3.5	5.58	Highly conserved
HSPG2	NC_000001.10:g.22150183 T > G	rs144864169	0.776	PP3	VUS	3.6	5.03	Highly conserved
LDLR	NC_000019.9:g.11221357 G > A	rs72658860	0.815	BP2, PP3, PP2, BA1, BS2, BP6	B	4.1	4.37	Highly conserved
LIPA	NC_000010.10:g.90982268 C > T	rs116928232	0.119	PM3, PP1, PS3, PM2, PP3, PP5	P	4.6	4.77	Highly conserved
LIPC	NC_000015.9:g.58834770 T > C	rs375115836	0.977	PP3, PM2	LP	4.9	5.08	Highly conserved
LRP1	NC_000012.11:g.57593052 A > G	rs1395154614	0.955	PP3, PM2, PP2	LP	4.6	4.98	Highly conserved
MSR1	NC_000008.10:g.16012594 G > A	rs41341748	—	PVS1, BA1, BS2, BP6	B	0.7	2.84	Low conservation
NPC1	NC_000018.9:g.21113475 T > C	rs35248744	0.844	PP3, PP2, BS1, BS2, BP6	B	5	5.54	Highly conserved
NPC1L1	NC_000007.13:g.44575482 A > T	rs79836534	0.869	PM2, PP3	VUS	4.1	4.23	Highly conserved
NR1H3	NC_000011.9:g.47282095 G > A	rs774079609	0.974	PP3, PM2	LP	5.5	5.08	Highly conserved
PCSK9	NC_000001.10:g.55505647 G > T	rs11591147	0.028	BA1, BS2, BP4, BP6	B	0.1	−0.263	Low conservation
PGM1	NC_000001.10:g.64120045 C > T	rs397515423	—	PM3, PVS1, PM2	P	3.3	4.51	Highly conserved
PHKA1	NC_000023.10:g.71802375 C > T	rs782546754	0.917	PM2, PP3	VUS	3.5	4.26	Highly conserved
PYGM	NC_000011.9:g.64521496 G > A	rs116135678	0.946	PM3, PP3, PM2, PP2, PP5	P	5.2	4.8	Highly conserved
SI	NC_000003.11:g.164739053 C > T	rs121912616	0.521	PM2, PP5	LP	5.1	4.64	Highly conserved
STAB2	NC_000012.11:g.104142911 G > A	rs375507356	0.87	PM2, PP3	VUS	5.8	5.29	Highly conserved

a The table lists 34 genetic variants distributed across 30 genes that passed a multi‐tiered filtering pipeline and segregate exclusively with one clinical phenotype. Variants in carbohydrate‐metabolism genes occur only in sCOVID‐19 cases, whereas those in cholesterol/lipoprotein‐metabolism genes are confined to MIS‐C cases, suggesting pathway‐specific genetic susceptibilities. Column definitions: Gene – HGNC symbol; HGVSG – HGVS genomic nomenclature; dbSNP – rsID; REVEL – predicted pathogenicity score; ACMG criteria – evidence codes assigned by Franklin (PS, PM, PP, BS, BP); ACMG classification – final tier (P, LP, VUS, LB, B); PhyloP and GERP++ – nucleotide conservation scores; Conservation class – “highly conserved” when PhyloP > 2 and GERP++ > 4.

Clinical and evolutionary annotations further underline the relevance of this 34 genetic variant panel. Under the ACMG/AMP framework, 14 genetic variants (41,2%) were classified as pathogenic or likely pathogenic, 13 (38,2%) as variants of uncertain significance, and 7 (20,6%) as benign. Conservation scores were available for 32 of the 34 genetic variants. Of these, 28 (87,5%; 82,4% of the full shortlist) satisfied the stringent dual threshold for evolutionary constraint (PhyloP > 2 and GERP++ RS > 4) and were labelled highly conserved. Only a single genetic variant met the PhyloP criterion without exceeding the GERP++ threshold and was therefore classified as conserved (PhyloP only), whereas three genetic variants failed to reach either cut‐off and were placed in the low‐conservation category. Although neither ACMG classification nor conservation metrics influenced genetic variant selection, the concordance between predicted clinical impact, strong evolutionary constraint, and phenotype‐specific distribution suggests that these genetic variants may contribute to the divergent pathophysiological processes underlying sCOVID‐19 and MIS‐C.

**Figure 6:**
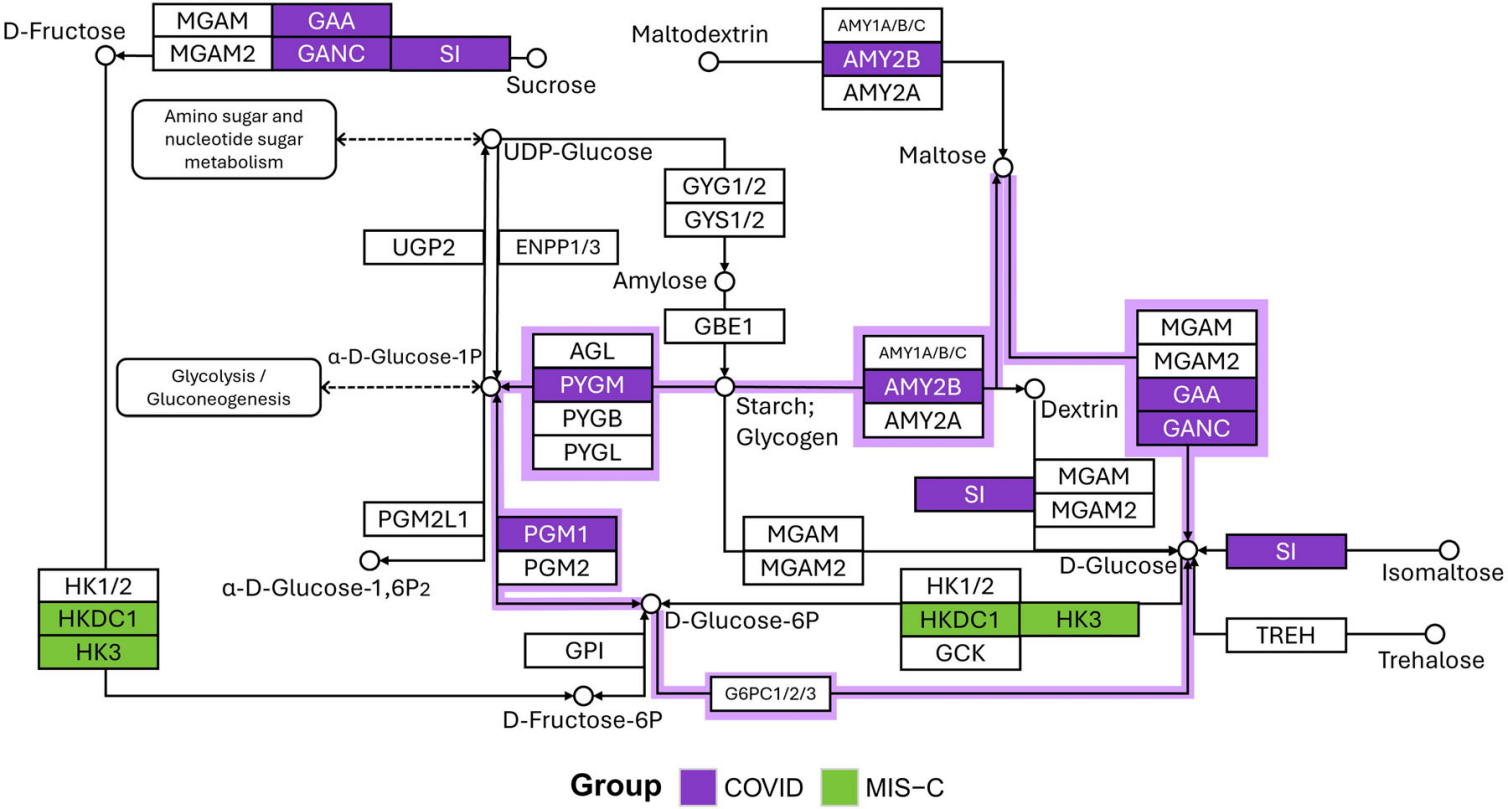
Carbohydrate metabolism and glycogen breakdown pathways. Adapted from the KEGG Starch and Sucrose Metabolism pathway, this diagram highlights key enzymes involved in carbohydrate metabolism as identified through over‐representation analysis (ORA). Genes harboring variants in sCOVID‐19 are shown in purple, whereas those in MIS‐C appear in green. Light purple outlines indicate components of the KEGG glycogen degradation pathways (N00718 and N00720), underscoring the prominent role of glycogenolysis in the pathophysiology of severe COVID‐19 and MIS‐C.

**Figure 7:**
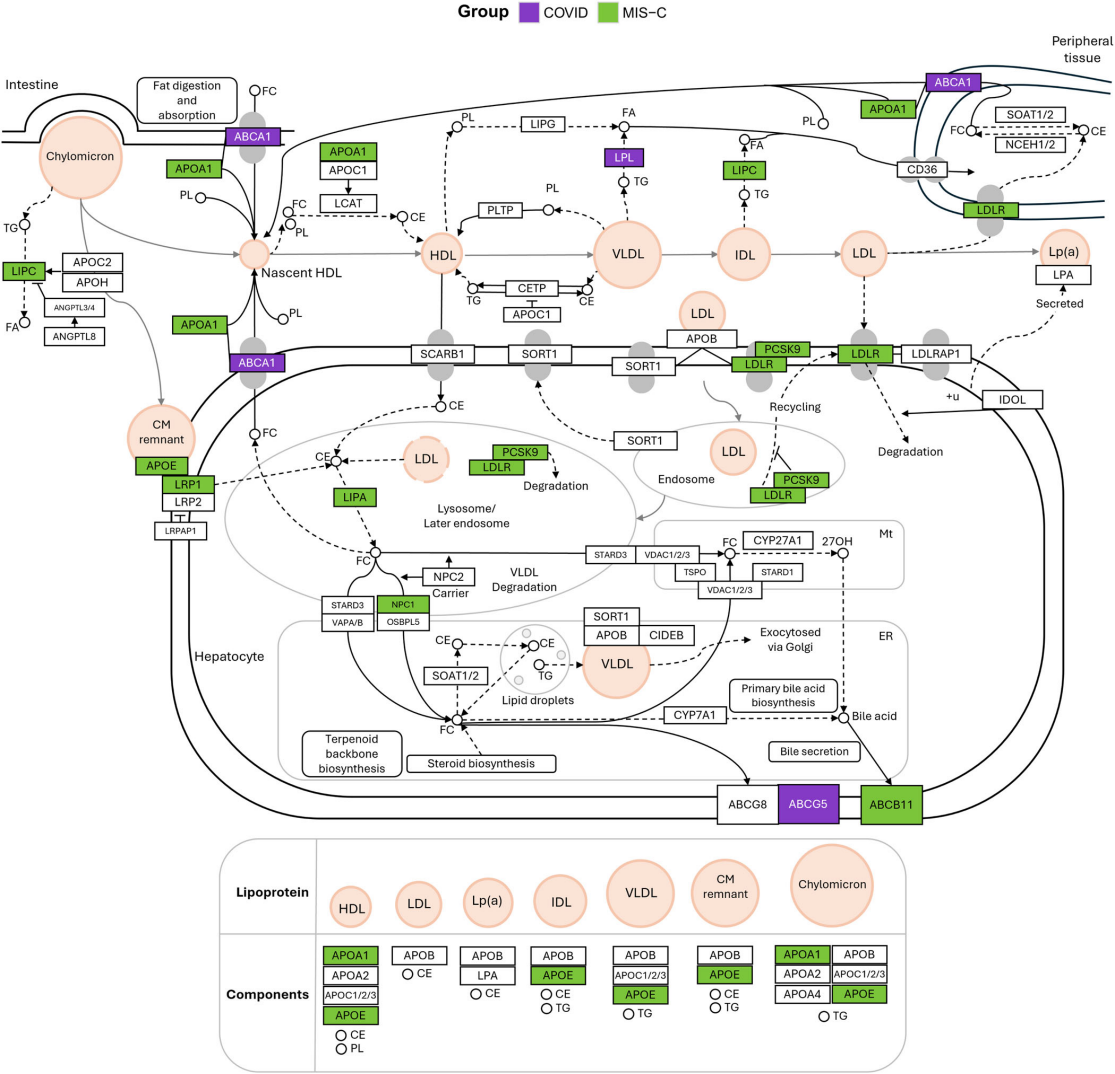
Cholesterol metabolism pathway. Adapted from the KEGG Cholesterol Metabolism pathway, this diagram highlights genes within the Cholesterol Metabolism supercategory identified by over‐representation analysis (ORA). Genes harboring variants in sCOVID‐19 are shown in purple, while those in MIS‐C appear in green. The pathway illustrates the interplay of lipoproteins – HDL, LDL, VLDL, and chylomicrons – in cholesterol transport, uptake, and metabolism, emphasizing processes that may be disrupted by potentially pathogenic genetic variants.

Within carbohydrate metabolism, KEGG analysis identified eight genes in the starch and sucrose metabolism pathway: six in sCOVID‐19 and two in MIS‐C (Figure 6). The six sCOVID‐19 genes clustered in the Glycogen degradation and Glycogen degradation (amylase) subnetworks, aligning with the enrichment of the Glycogen breakdown (glycogenolysis) pathway in Reactome (Figure 3b). Conversely, KEGG analysis of cholesterol metabolism analysis revealed 12 genes involved in cholesterol and lipoprotein metabolism, nine of which were detected in the MIS‐C group (Figure 7).

### 3.6 Three‐Dimensional Protein Modeling of Potentially Pathogenic Genetic Variants in Carbohydrate Metabolism and Cholesterol Metabolism

**Figure 8:**
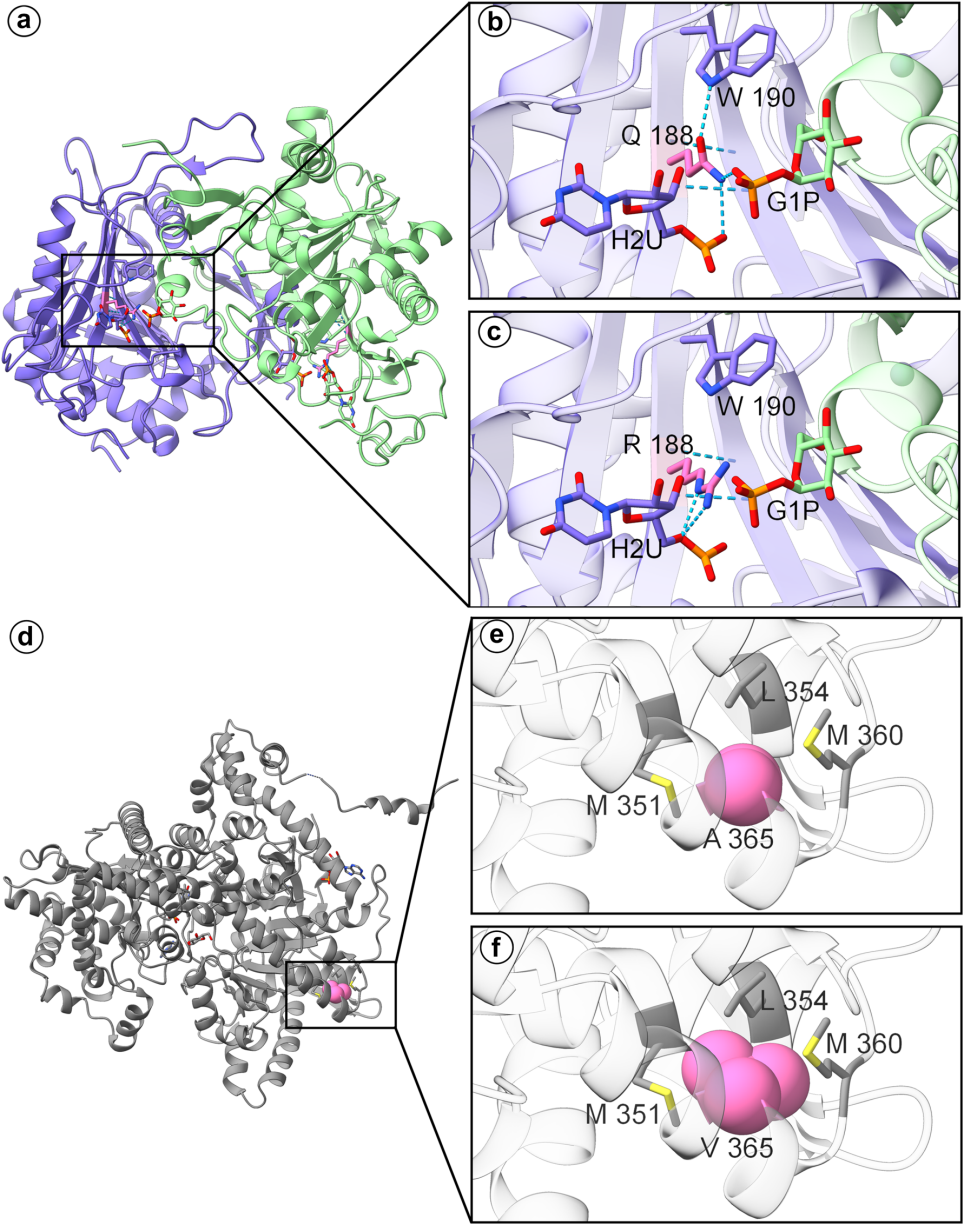
Structural consequences of variants in carbohydrate metabolism genes. In silico analyses were conducted to model the effects of missense variants on protein conformation and function, focusing on key residues and potential disruptions in active sites or binding pockets: (a–c) GALT and NP_000146.2:p. Gln188Arg (rs75391579): (a) GALT structure. (b) Active site close‐up: Gln188 bonding with Trp190, G1P, and H2U. (c) Variant model: p. Gln188Arg disrupts Trp190 and ligand interactions, likely reducing catalytic activity. (d–f) PYGM and NP_005600.1:p. Ala365Val (rs116135678): (d) PYGM structure with AMP and glucose. (e) Close‐up of Ala365 (pink). (f) Variant model: p.Ala365Val causes steric clashes with Met351, Met360, and Leu354.

**Figure 9:**
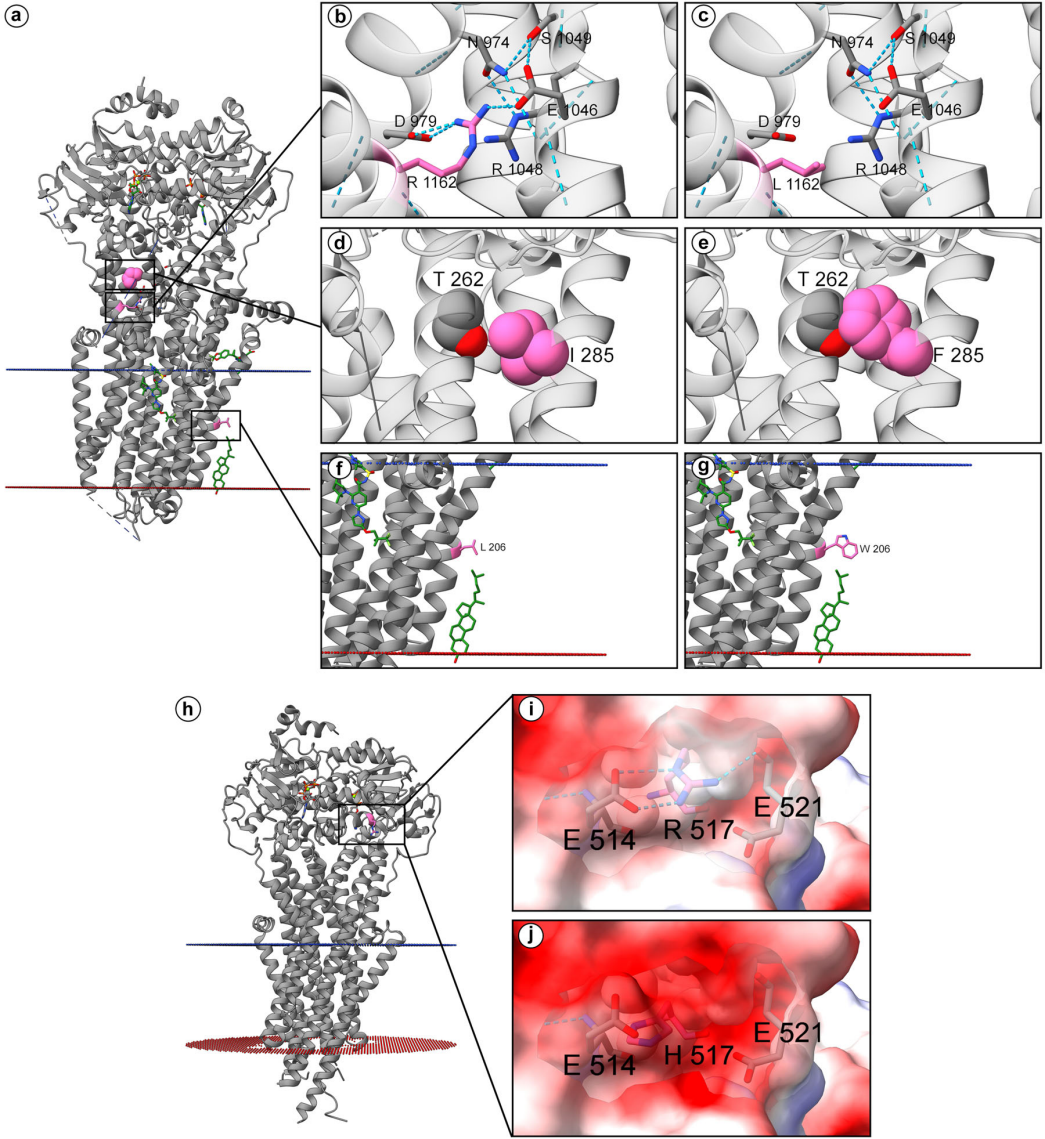
Structural consequences of variants in cholesterol metabolism genes. In silico analyses were conducted to model the effects of missense variants on protein conformation and function, focusing on key residues and potential disruptions in active sites or binding pockets. (a–g) CFTR chloride channel and NP_000483.3:p.[Arg1162Leu; Ile285Phe; Leu206Trp]: (a) CFTR with predicted membrane orientation. (b) Close‐up of Arg1162, which forms hydrogen bonds with Asp979 and Glu1046. (c) Variant model: p. Arg1162Leu (rs1800120) disrupts these hydrogen bonds. (d) Close‐up of Ile285. (e) Variant model: p. Ile285Phe (rs151073129) introduces steric bulk, potentially clashing with Thr262. (f) Close‐up of Leu206 near cholesterol (CLR). (g) Variant model: p. Leu206Trp (rs121908752) may perturb protein‐solvent and membrane interactions. All models depict extracellular surfaces in red and intracellular surfaces in blue. (h–j) ABCB11 and NP_003733.2:p.Arg517His: (h) Overall structure of the bile salt export pump ABCB11. (i) Detailed view of Arg517, which forms hydrogen bonds with Glu514 and Glu521 within the nucleotide‐binding region. (j) Variant model: p.Arg517His disrupts both stabilizing hydrogen bond networks. Electrostatic potential surfaces color scheme demonstrates the transitions from neutral to negative charge distribution upon aminoacidic substitution: electronegative regions in red, electropositive regions in blue, and neutral areas in white.

To provide a comprehensive understanding of the structural impact of genetic variants, they were subjected to three‐dimensional protein modeling. These analyses reveal how amino‐acid substitutions can impair protein stability, diminish catalytic activity, or disrupt ligand‐binding networks. From this extensive analysis, four key genetic variants were selected for detailed presentation and in‐depth discussion in the main text (Figures 8 and 9), based on three stringent filters: a high pathogenicity prediction (REVEL > 0.80), an ACMG/AMP classification of pathogenic, and strong evolutionary constraint (PhyloP > 4.0 together with GERP++ > 4.5). Two arise in carbohydrate‐metabolism genes: GALT p.Gln188 Arg (rs75391579) and PYGM p.Ala365Val (rs116135678). The remaining two genetic variants identified are involved in cholesterol and lipoprotein metabolism genes: CFTR p.Leu206Trp (rs121908752) and ABCB11 p.Arg517His (rs760750012). For CFTR protein, beyond the p.Leu206Trp variant, other missense variants identified (Arg1162Leu and Ile285Phe) were also structurally analyzed, with details provided for all three missense variants in Figure 9. The CFTR p.Phe508del variant was identified but not amenable to this specific structural modeling approach due to the method limitation to work with sequence deletions. Additional structural modeling for an expanded panel of carbohydrate‐metabolism genes (AMY2B, GAA, GALE, GANC, PGM1, PHKA1, SI) and cholesterol‐metabolism genes (ABCA5, ABCB4, ADIPOQ, APOE, NR1H3) was likewise performed and is provided in Supplementary Results. The LRP1 p.Tyr3245Cys variant could not be structurally characterized due to the protein′s substantial molecular weight (4,544 amino acids) and absence of experimental structures encompassing the variant site, though the Tyr‐to‐Cys substitution potentially affects disulfide bonding patterns within the extracellular domain. LIPA was excluded from the structural‐modelling section because the interrogated variant, p.Gln298= is synonymous. In LIPC, the p.Ile165Thr substitution proved structurally neutral: situated in a buried core region, the threonine side chain introduced no significant steric, electrostatic, or cavity‐volume changes, rendering additional modelling results non‐informative.

*GALT* encodes the enzyme galactose‐1‐phosphate uridylyltransferase, which catalyzes a key step in the metabolism of galactose by converting galactose‐1‐phosphate and UDP‐glucose to glucose‐1‐phosphate and UDP‐galactose (Figure 8a). A variant designated NP_000146.2:p.Gln188Arg (rs75391579) has been identified in GALT and is associated with a severe form of galactosemia, a metabolic disorder characterized by the impaired ability to metabolize galactose. The p.Gln188Arg variant involves the substitution of the medium‐sized polar glutamine residue with the larger, basic arginine residue within the active site of the enzyme. Structural analysis revealed that the wild‐type residue Gln188 forms crucial hydrogen bond interactions with Trp190 and the ligands 1‐O‐phosphono‐α‐d‐glucopyranose (G1P) and 5,6‐dihydrouridine‐5′‐monophosphate (H2U) (Figure 8b). However, the p.Gln188Arg variant is predicted to disrupt these interactions due to the introduction of the larger, positively charged arginine side chain (Figure 8c). The loss of hydrogen bonding interactions with Trp190 and the ligands is anticipated to impair the proper folding and stability of the enzyme, with potential to reduce its catalytic activity.

*PYGM* encodes the glycogen phosphorylase muscle form, a crucial enzyme involved in the breakdown of glycogen, the primary storage form of glucose in skeletal muscle (Figure 8d). Structural analysis of PYGM revealed that the NP_005600.1:p.Ala365Val (rs116135678) missense variant could result in significant steric hindrance. The substitution of the small, non‐bulky alanine residue at position 365 (Figure 8e) with the larger, branched‐chain valine side chain is predicted to cause clashes with the neighboring residues Met351, Met360, and Leu354 (Figure 8f). These steric conflicts arising from introducing the bulkier valine residue could disrupt the precise packing and folding of the local structural environment, potentially destabilizing the overall tertiary structure and conformational dynamics of the glycogen phosphorylase muscle form.

*CFTR* encodes a chloride channel implicated in cystic fibrosis pathogenesis and cholesterol trafficking. Structural examination revealed that the wild‐type residue Arg1162 forms hydrogen bonds with Asp979 and Glu1046 on adjacent α‐helices. Moreover, Glu1046 participates in a hydrogen bonding network involving Ser1049, which connects to Asn974 and Arg1048. (Figure 9b). The substitution of the positively charged Arg1162 with the nonpolar leucine (NP_000483.3:p.Arg1162Leu, rs1800120) would disrupt these critical hydrogen bonds with Asp979 and Glu1046, potentially destabilizing and impairing CFTR function (Figure 9c). The NP_000483.3:p.Ile285Phe (rs151073129) variant occurs within an α‐helix of CFTR (Figure 9d). Substituting the smaller isoleucine with the larger, aromatic phenylalanine results in increased steric bulk, potentially clashing with Thr262on an adjacent helix (Figure 9e). Such steric hindrance can disrupt the structural integrity and stability of the adjacent helix, affecting CFTR′s overall tertiary structure and potentially perturbing interactions with essential biomolecules. The NP_000483.3:p.Leu206Trp (rs121908752) variant is situated within the transmembrane region of CFTR, facing the solvent‐exposed region near a cholesterol molecule (CLR) observed in the electron microscopy structure (PDB ID: 8EIO) (Figure 9f). The introduction of the bulky, aromatic tryptophan residue in place of the smaller, aliphatic leucine could significantly influence protein–solvent interactions, disrupt membrane integrity, and modulate the dynamics of protein–membrane associations (Figure 9g).

*ABCB11* encodes the bile salt export pump (BSEP), an ATP‐dependent transporter whose efflux of hydrophobic bile acids preserves hepatic bile acid balance and, via bile salt–dependent biliary lipid secretion, integrates into systemic lipid metabolism. The variant NP_003733.2:p.Arg517His, located within the highly conserved nucleotide‐binding fold region essential for ATP binding and hydrolysis, demonstrated potential structural perturbations. Structural analysis revealed that the wild‐type Arg517 residue establishes hydrogen bond interactions with both Glu514 and Glu521, creating a stabilizing network within the ATP‐binding domain (Figure 9i). The substitution of the positively charged arginine with the smaller histidine residue disrupts both hydrogen bonding interactions simultaneously (Figure 9j), while electrostatic potential mapping demonstrated a transition from neutral to negative charge distribution at the variant site (Figure 9h,i), potentially compromising ATP binding affinity and nucleotide hydrolysis efficiency.

## 4 Discussion

ORA revealed distinct gene sets in sCOVID‐19 and MIS‐C. A combined analysis of all samples showed enrichment of cholesterol and lipoprotein metabolism, but after stratification only the MIS‐C group retained these pathways, whereas carbohydrate metabolism—particularly glycogen breakdown—was uniquely enriched in sCOVID‐19.

The ACMG/AMP classification and evolutionary metrics converge on a coherent signal of functional relevance of the genetic variants identified in our study. Fourteen of the 34 shortlisted genetic variants (41.2%) meet ACMG/AMP criteria for pathogenicity or likely pathogenicity, 13 (38.2%) are genetic variants of uncertain significance, and 7 (20.6%) are benign or likely benign. Conservation scores are available for 32 genetic variants; 28 (87.5%) lie at residues under strong purifying selection. Kinship analysis confirmed that none of the 44 participants are related at the third‐degree level or closer, indicating that these concordant annotations are not driven by familial clustering and that genotype–phenotype comparisons represent independent observations.

Although SARS‐CoV‐2 infection is an environmental trigger, genome‐wide and exomic surveys have uncovered multiple genomic regions that measurably influence both susceptibility to infection and the clinical course of COVID‐19. These loci account for only a fraction of overall risk and therefore cannot yet support routine individual stratification, but they already sharpen mechanistic insight and highlight candidate targets for diagnostic and therapeutic development [38].

Because the ACMG/AMP framework was developed for highly penetrant Mendelian disorders, its categories should be interpreted here as indicators of potential functional effect rather than definitive causal pathogenicity. Variants labelled pathogenic may act mainly as risk modifiers in these multifactorial settings, whereas several VUS situated at highly conserved residues could contribute to disease only in combination with additional genetic or environmental factors. Recent proposals to adapt ACMG terminology for complex traits by introducing “predisposing” tiers and explicitly incorporating polygenic background reinforce this interpretation [39]. Accordingly, the convergence of ACMG and structural modeling evidence, strong evolutionary constraint, and phenotype‐specific distribution suggests that the present variant set may contribute to the divergent pathophysiological processes underlying MIS‐C and severe COVID‐19.

In sCOVID‐19, three enrichment categories not observed in MIS‐C were identified: cardiomyopathies, carbohydrate metabolism, and serine‐type peptidase activity. This finding aligns with the known impact of pre‐existing heart conditions on COVID‐19 outcomes, since the infection itself can exacerbate myocarditis and other cardiovascular complications [40]. Moreover, a study comparing shared differentially expressed genes in heart failure and COVID‐19 patients found the term “starch and sucrose metabolism” enriched [41], suggesting the relevance of these pathways. Consistently, among the 12 carbohydrate metabolism genes associated with sCOVID‐19, 8 participate in starch and sucrose metabolism (KEGG), primarily contributing to glycogen breakdown (Figure 6).

Carbohydrate metabolism is pivotal for immune cell function. Activated mucosal‐associated invariant T (MAIT) cells utilize glycogen for rapid cytotoxic responses [42]. Dendritic cells require glycogen for maturation and lymphocyte activation, as blocking glycogen metabolism impairs these processes. Furthermore, dendritic cells use distinct pathways for carbon derived from glucose versus glycogen, with glycogen‐derived carbon preferentially contributing to citrate synthesis, which supports cytokine production and secretion [43, 44]. In CD8+ T memory cells, glucose and glycogen metabolism are specifically orchestrated and compartmentalized to ensure cell function. These cells show positive regulation of glycogenesis via gluconeogenesis. The resulting glycogen is then channeled into glycogenolysis, increasing levels of glucose‐6‐phosphate (G6P). G6P derived from glycogen breakdown is oxidized in the pentose phosphate pathway (PPP), generating NADPH and high levels of reduced glutathione in these cells. Disruption of this pathway leads to an accumulation of reactive oxygen species, impairing CD8+ T cell formation and infection clearance [45, 46]. Similarly, macrophages rely on the same glycogen breakdown pathway to supply NADPH and glutathione [47], while neutrophils in severe COVID‐19 exhibit elevated glycolysis, cytoplasmic glycogen accumulation, and enhanced glycogenolysis [48].

The data presented here point to a significant role for glycogenolysis in immune responses during SARS‐CoV‐2 infection. Two mechanisms may explain its importance: (i) glycogen breakdown produces 3 ATP per glucose, outpacing the 2 ATP from glycolysis, thereby meeting heightened energy demands, and (ii) the partial diversion of G6P into PPP generates NADPH, sustaining glutathione levels under oxidative stress. Both mechanisms provided by glycogen breakdown could contribute to immune cell survival during SARS‐CoV‐2 infection. Additionally, genetic variations in two of the four enzymes of the Leloir pathway, GALT and GALE, which feed into glycogenesis, may further compromise energy production via glycolysis, energy storage via glycogenesis, and redox balance in infected children.

In MIS‐C, the identified potentially pathogenic variants may functionally affect processes related to cholesterol and lipoprotein metabolism (Figures 3b, 5, and 7). This hypothesis aligns with reports that low levels of total and HDL cholesterol (HDL‐C) correlate with increased hospitalization risk in COVID‐19 and other infections [49]. Indeed, apolipoprotein A‐1, a key component of HDL‐C, has been linked to antiviral activity, and its anti‐inflammatory and immunomodulatory roles have been discussed [50]. Studies have demonstrated that cholesterol levels are lower in patients infected with SARS‐CoV‐2 compared to healthy individuals, and as the severity of COVID‐19 increases, cholesterol levels decrease [51]. Transcriptomic data showed that the expression of genes involved in the cholesterol synthesis pathway is inhibited during SARS‐CoV‐2 infection [52]. Furthermore, plasma proteomic analyses in children with COVID‐19 and MIS‐C also indicate suppression of cholesterol and lipoprotein metabolism pathways [53]. Notably, pediatric COVID‐19 cases presenting low HDL‐C develop more severe symptoms, with particularly low HDL‐C levels seen in severe cases that developed MIS‐C [54]. In these pediatric COVID‐19 patients, a decrease in cholesterol efflux capacity of macrophages was found, which was also associated with the increased severity of COVID‐19 and MIS‐C [54].

Genetic variants that disrupt cholesterol transport may contribute to reduced HDL levels and subsequent cholesterol accumulation in cell membranes [55]. These cholesterol‐rich membrane domains, or lipid rafts, host ACE2 and other receptors critical for SARS‐CoV‐2 entry. Elevated membrane cholesterol can reorganize ACE2 within GM1 clusters, thereby enhancing viral binding and endocytosis. Consequently, pharmacological approaches aimed at disrupting GM1 clusters or promoting cholesterol efflux may offer therapeutic advantages for critically ill COVID‐19 patients [56]. HDL itself integrates innate and adaptive immunity by regulating lipid raft cholesterol levels, influencing toll‐like receptor function and B‐/T‐cell receptor activity. In animal models, disruptions in HDL metabolism or cholesterol efflux produce phenotypic traits consistent with immune disorders [57]. These observations suggest that genetic variants in cholesterol‐ and lipoprotein‐related genes could underlie the low cholesterol phenotypes observed in MIS‐C and intensify inflammatory responses, given that defective cholesterol efflux in dendritic cells and macrophages promotes hyperinflammatory states [58, 59].

Structural modeling of these carbohydrate‐ and cholesterol‐related genetic variants suggests that most substitutions are likely to compromise protein function, consistent with their predicted or documented pathogenicity. Additional studies are necessary to clarify the roles of these genetic changes in disease severity.

Some study limitations merit consideration. First, the cohort comprised only 15 children with severe COVID‐19 and 29 with MIS‐C, a sample size that offers little power for single genetic variant association tests. Recruitment of pediatric cases with these rare and acute phenotypes is intrinsically difficult, and many reports on comparable conditions present similar number of patients [15, 53, 60]. To maximize biological insight, ORA was applied. By collapsing rare, putatively deleterious genetic variants to the gene level and testing pathway enrichment, ORA converts thousands of sparse variant‐level comparisons into a few hundred pathway‐level hypotheses, greatly easing the multiple‐testing burden. Because the hypergeometric statistic that underlies ORA depends chiefly on the proportion of genes in the list rather than on sample count, its statistical power is largely preserved even when a gene presents a variant in only one individual [61]. Support for this strategy arise from a plasma‐proteomics study of paediatric MIS‐C and COVID‐19‐related ARDS, in which ORA applied to differentially abundant proteins per group still recovered cholesterol‐related pathways – despite the small data set. Consistent with that precedent, ORA of the present WES data highlighted carbohydrate‐metabolism pathways in severe COVID‐19 and cholesterol/lipoprotein pathways in MIS‐C – signals that single‐gene tests could not detect at the current sample size. ORA therefore serves as a biologically informed signal amplifier that mitigates the constraints imposed by the cohort size. Even so, WES coupled with ORA captures only part of the genetic architecture of these conditions. Larger cohorts, integration of complementary omics layers and functional validation will be needed to refine the present findings.

In conclusion, whole‐exome sequencing and over‐representation analyses identified distinct genetic variants in pediatric severe COVID‐19 and MIS‐C. Carbohydrate metabolism genes, particularly those involved in glycogen breakdown, were predominantly affected in sCOVID‐19, whereas MIS‐C exhibited variants linked to cholesterol and lipoprotein metabolism. These findings expand current understanding of the genetic landscape in severe pediatric COVID‐19 outcomes and may inform future diagnostic and therapeutic strategies.

## Author Contributions

Conceptualization: Fabio Passetti, Hellen Geremias dosSantos, Helisson Faoro, Zilton Farias Meira deVasconcelos, Patricia Savio deAraujo‐Souza, Jeanine Marie Nardin, Acácia Maria Lourenço Francisco Nasr, Luiz Lehmann Coutinho, Benilton de Sá Carvalho, Rubens Cat, Fabricio Klerynton Marchini, Luis Gustavo Morello. Methodology: Alysson Henrique Urbanski, Flávia Cristina de PaulaFreitas, Tiago Minuzzi Freire da Fontoura Gomes, Michelle Orane Schemberger, Lucas de Almeida Machado, Deborah Antunes dos Santos, Benilton de Sá Carvalho, Luiz Lehmann Coutinho, Acácia Maria Lourenço Francisco Nasr, Irina Nastassja Riediger, Jeanine Marie Nardin, Ana Carolina Ramos Guimarães, Helisson Faoro, Hellen Geremias dos Santos, Fabio Passetti. Software: Alysson Henrique Urbanski, Flávia Cristina de Paula Freitas, Tiago Minuzzi Freire da Fontoura Gomes, Letícia Graziela Costa Santos, Esdras Matheus Gomes da Silva, Vinícius Da Silva Coutinho Parreira, Hellen Geremias dos Santos. Validation: Michelle Orane Schemberger. Formal analysis: Alysson Henrique Urbanski, Flávia Cristina de Paula Freitas, Tiago Minuzzi Freire da Fontoura Gomes, Michelle Orane Schemberger, Lucas de Almeida Machado, Deborah Antunes dos Santos, Hellen Geremias dos Santos. Investigation: Alysson Henrique Urbanski, Flávia Cristina de Paula Freitas, Tiago Minuzzi Freire da Fontoura Gomes, Michelle Orane Schemberger, Bárbara Carvalho Santos dos Reis, Lucas de Almeida Machado, Deborah Antunes dos Santos, Rubens Cat, Benilton de Sá Carvalho, Marcus F. Oliveira, Ana Carolina Ramos Guimarães, Patricia Savio de Araujo‐Souza, Zilton Farias Meira de Vasconcelos, Helisson Faoro, Hellen Geremias dos Santos, Fabio Passetti. Resources: Bárbara Carvalho Santos dos Reis, Flavia Amêndola Anísio de Carvalho, Roberta Soares Faccion, Daniela Prado Cunha, Margarida dos Santos Salú, Daniella Campelo Batalha Cox Moore, Mayra Marinho Presibella, Juliana Fontes Noguchi, Henrique Lira Borges, Lais Kimie Tomiura, Carmen Australia Paredes Marcondes Ribas, Luiza Silva de Castro, Maria Regina Tizzot, Mauricio Marcondes Ribas, Gilberto Pascolat, Liya Regina Mikami, Arnaldo Prata‐Barbosa, Zilton Farias Meira de Vasconcelos, Fabio Passetti. Data curation: Alysson Henrique Urbanski, Flávia Cristina de Paula Freitas, Tiago Minuzzi Freire da Fontoura Gomes, Michelle Orane Schemberger, Lucas de Almeida Machado, Deborah Antunes dos Santos, Hellen Geremias dos Santos. Writing – original draft preparation: Alysson Henrique Urbanski. Writing – review and editing: Alysson Henrique Urbanski, Flávia Cristina de Paula Freitas, Tiago Minuzzi Freire da Fontoura Gomes, Michelle Orane Schemberger, Bárbara Carvalho Santos dos Reis, Flavia Amêndola Anísio de Carvalho, Roberta Soares Faccion, Lucas de Almeida Machado, Deborah Antunes dos Santos, Mayra Marinho Presibella, Juliana Fontes Noguchi, Henrique Lira Borges, Lais Kimie Tomiura, Luiza Silva de Castro, Letícia Graziela Costa Santos, Esdras Matheus Gomes da Silva, Vinícius Da Silva Coutinho Parreira, Luis Gustavo Morello, Fabricio Klerynton Marchini, Maria Regina Tizzot, Mauricio Marcondes Ribas, Gilberto Pascolat, Fábio Fernandes da Rocha Vicente, Alexandre Rossi Paschoal, Rubens Cat, Arnaldo Prata‐Barbosa, Benilton de Sá Carvalho, Jaqueline Carvalho de Oliveira, Marcus F. Oliveira, Luiz Lehmann Coutinho, Acácia Maria Lourenço Francisco Nasr, Irina Nastassja Riediger, Jeanine Marie Nardin, Liya Regina Mikami, Ana Carolina Ramos Guimarães, Patricia Savio de Araujo‐Souza, Zilton Farias Meira de Vasconcelos, Helisson Faoro, Hellen Geremias dos Santos, Fabio Passetti. Visualization: Alysson Henrique Urbanski, Flávia Cristina de Paula Freitas, Tiago Minuzzi Freire da Fontoura Gomes, Michelle Orane Schemberger, Bárbara Carvalho Santos dos Reis, Lucas de Almeida Machado, Deborah Antunes dos Santos, Ana Carolina Ramos Guimarães, Patricia Savio de Araujo‐Souza, Zilton Farias Meira de Vasconcelos, Helisson Faoro, Hellen Geremias dos Santos, Fabio Passetti. Supervision: Fabio Passetti; Project administration: Fabio Passetti; Funding acquisition: Fábio Fernandes da Rocha Vicente, Alexandre Rossi Paschoal, Zilton Farias Meira de Vasconcelos, Helisson Faoro, Fabio Passetti.

## Conflicts of Interest

The authors declare no conflicts of interest.

## Supporting information


Supplementary Data 1.

Supplementary Data 2.

**Supplementary Figure 1:** Comparison of age distribution between sCOVID‐19 and MIS‐C pediatric patients. **Supplementary Figure 2:** Whole‐exome sequencing coverage density distribution in sCOVID‐19 and MIS‐C samples. **Supplementary Figure 3:** Expanded structural consequences of variants in carbohydrate metabolism genes. **Supplementary Figure 4:** Expanded structural consequences of variants in cholesterol/lipoprotein metabolism genes.

## Data Availability

The data that support the findings of this study are openly available in National Center for Biotechnology Information (NCBI) at https://www.ncbi.nlm.nih.gov/bioproject/PRJNA1147596, reference number PRJNA1147596. All whole‐exome sequencing data have been deposited in the National Center for Biotechnology Information (NCBI) BioProject database under accession number PRJNA1147596 [62].
