# Supplementary material for: Genetic Variants Affect Distinct Metabolic Pathways in Pediatric Multisystem Inflammatory Syndrome and Severe COVID‐19
Source: J Med Virol. 2025 Aug 16;97(8):e70556. doi: 10.1002/jmv.70556 (PMC12357531; doi:10.1002/jmv.70556)
Supplement: Supplementary file 3 — Supplementary Figure 1: Comparison of age distribution between sCOVID‐19 and MIS‐C pediatric patients. Supplementary Figure 2: Whole‐exome sequencing coverage density distribution in sCOVID‐19 and MIS‐C samples. Supplementary Figure 3: Expanded structural consequences of variants in carbohydrate metabolism genes. Supplementary Figure 4: Expanded structural consequences of variants in cholesterol/lipoprotein metabolism genes. [file JMV-97-e70556-s002.docx]

#
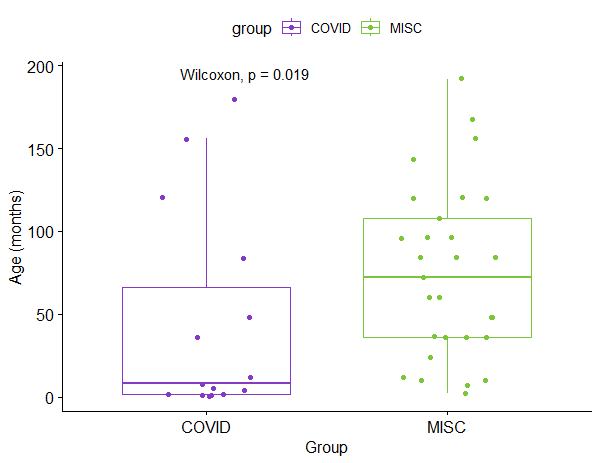


**Supplementary Figure 1: Comparison of age distribution between sCOVID-19 and MIS-C pediatric patients.**

# The box plots display the age distribution in months for pediatric patients diagnosed with sCOVID-19 (purple) and MIS-C (green). The median age, interquartile range, and individual data points are shown for each group. Wilcoxon test *p*-value is shown.

**
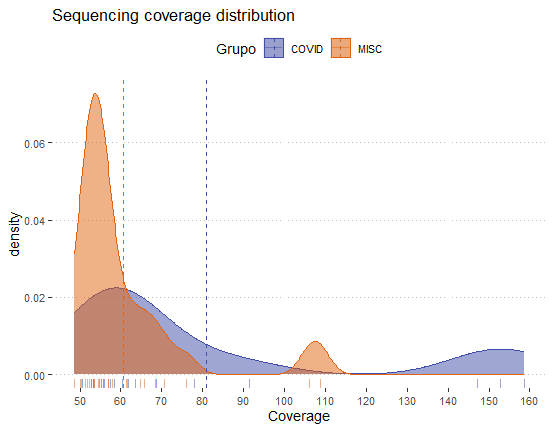
**

**Supplementary Figure 2: Whole-exome sequencing coverage density distribution in sCOVID-19 and MIS-C samples.**

The density plot illustrates the coverage distribution for samples from sCOVID-19 (blue) and MIS-C (orange) groups. The x-axis represents the coverage values, while the y-axis indicates the density. Vertical dashed lines mark the mean coverage for each group.

1. **Supplementary Methods**
   1. **Expanded Protein Structural Modeling**

To broaden the structural survey beyond the four variants discussed in the main text, a selected set of additional missense substitutions in carbohydrate and cholesterol/lipoprotein metabolism genes underwent an “expanded” structural-modelling workflow. The structural models were obtained from publicly available repositories as follows: AlphaFoldDB provided models for ABCA5 (Q8WWZ7), full-length ADIPOQ (Q15848), APOE (P02649), AMY2B (P19961), GANC (Q8TET4), PHKA1 (P46020) and SI (P14410). Experimentally determined structures were downloaded from the Protein Data Bank, namely the EM structure of ABCB4 (PDB 6S7P, 3.20 Å), the crystallographic trimeric globular domain of ADIPOQ (PDB 6U66, 0.99 Å), the GAA–acarbose complex (PDB 5NN8, 2.45 Å), the GALE–NAD⁺ complex (PDB 1EK5, 1.80 Å) and the PGM1–1-O-phosphono-α-D-glucopyranose complex (PDB 6SNO, 2.70 Å). Finally, an NR1H3 homology model was generated with SWISS-Model using the liganded human RXR-α/LXR-β heterodimer bound to DNA (template PDB 4NQA; 73.7 % sequence identity, 81 % coverage).

1. **Supplementary Results**
   1. **Expanded Protein Structural Modeling**

Structural modelling was extended to 12 additional coding variants, 7 in carbohydrate-metabolising enzymes and 5 in cholesterol/lipoprotein regulators, thereby complementing the four variants examined in the main text. The analyses identified three principal classes of structural perturbation: (i) steric clashes or cavity occlusion within catalytic cores, (ii) disruption of hydrogen-bond and salt-bridge networks essential for multimeric or transmembrane stability, and (iii) electrostatic or volumetric shifts at ligand- or DNA-binding interfaces. Variant-specific interpretations are presented below and illustrated in Supplementary Figures 3-4.


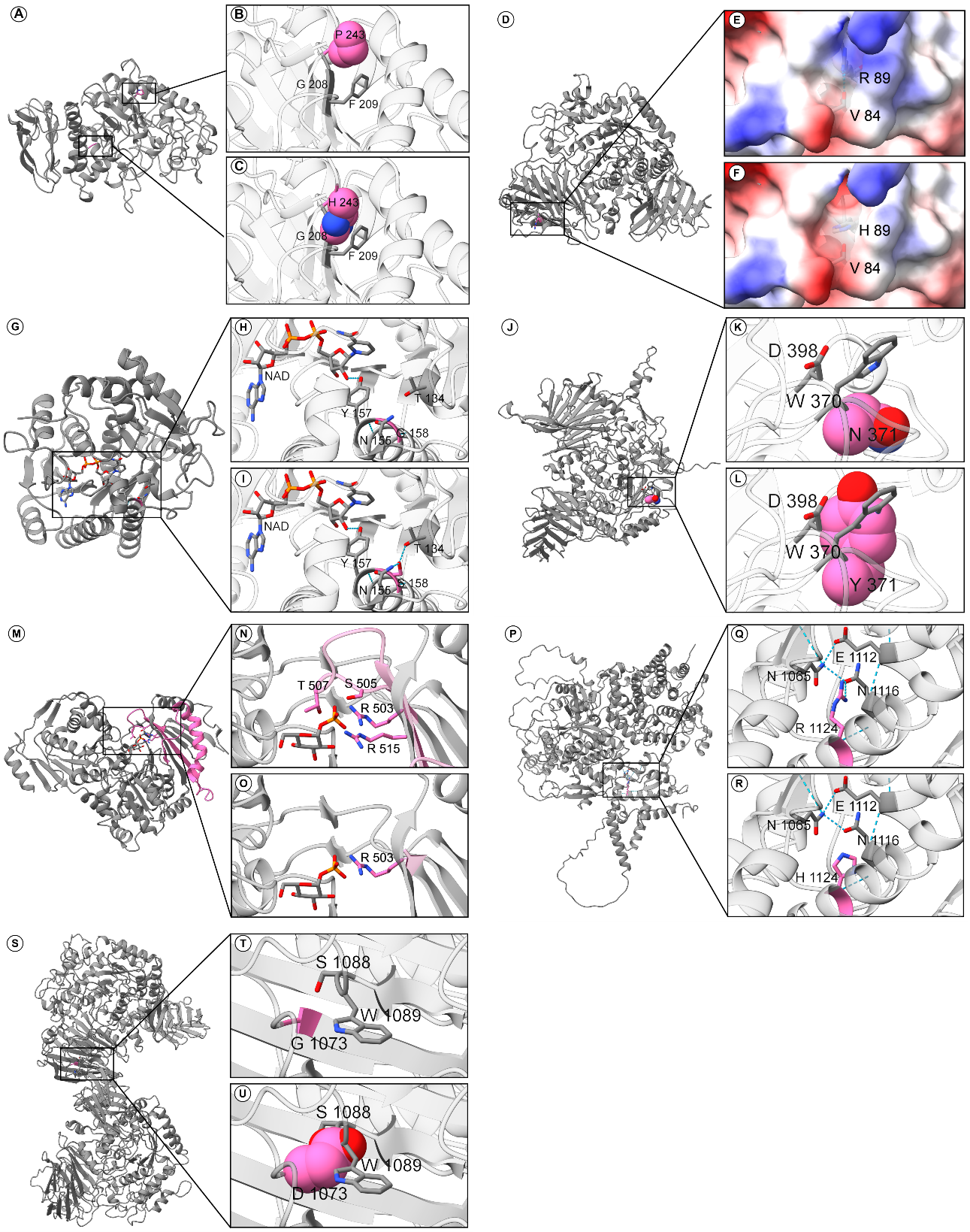


**Supplementary Figure 3. Expanded structural consequences of variants in carbohydrate metabolism genes.**

*In silico* analyses were conducted to model the effects of missense variants on protein conformation and function, focusing on key residues and potential disruptions in active sites or binding pockets:

**(A-C)** AMY2B and NP_066188.1:p.Pro243His: (A) AMY2B model. (B) Close-up of Pro243His (rs137860562). (C) Variant model: Pro243His induces steric clashes with Gly208 and Phe209, destabilizing the enzyme’s tertiary structure.

**(D-F)** GAA and NP_000143.2:p.Arg89His: (D) GAA model. (E) Close-up of Arg89His demonstrating hydrogen bond formation with the backbone oxygen of Val84. (F) Variant model: Arg89His eliminates the critical hydrogen bond interaction. Electrostatic potential mapping reveals a shift from positive (blue) to negative (red) charge distribution, with neutral regions depicted in white.

**(G-I)** GALE and NP_000394:p.Gly158Ser (rs374106365): (G) GALE structure with NAD⁺. (H) Active site close-up: Gly158 near Tyr157, which bonds with NAD⁺. (I) Variant model: p.Gly158Ser introduces new hydrogen bonds with Thr134 and Asn155, modifying the active site.

**(J-L)** GANC and NP_937784.2:p.Asn371Tyr (rs751368632): (J) GANC model. (K) Close-up of Asn371 (pink). (L) Variant model: p.Asn371Tyr induces steric clashes with Asp398 and Trp370.

**(M-O)** PGM1 and NP_001166289.1:p.Arg503*: (M) PGM1 model. (N) Close-up of Arg503 and critical phosphate-coordinating residues Ser505, Thr507, and Arg515. (O) Variant model: structural representation of the truncated protein showing complete loss of domain 4 and associated residues, resulting in catalytical and structural instability.

**(P-R)** PHKA1 and NP_002628.2:p.Arg1124His (rs782546754): (P) PHKA1 model. (Q) Close-up of Arg1124 hydrogen bond network. (R) Variant model: p.Arg1124His disrupts hydrogen bonds with Asn1116, destabilizing the network with Asn1065 and Glu1112, leading to structural perturbation.

**(S-U)** Sucrase-isomaltase (SI) and NP_001032.2:p.Gly1073Asp (rs121912616): (S) SI model. (T) Close-up of Gly1073. (U) Variant model: p.Gly1073Asp causes steric hindrance with Ser1088 and Trp1089.

*AMY2B* encodes the alpha-amylase 2B enzyme, which plays a crucial role in the breakdown of dietary starch and glycogen (Supplementary Figure 3A). Structural analysis revealed that the NP_066188.1:p.Pro243His (rs137860562) variant in AMY2B could result in significant steric hindrance. The introduction of the bulky histidine residue at position 243, in place of the smaller proline (Supplementary Figure 3B), is anticipated to cause clashes with the adjacent residues Gly208 and Phe209, which are located on a neighboring beta-sheet (Supplementary Figure 3C). Such steric clashes could disrupt the precise packing and folding of the beta-sheet region, potentially destabilizing the overall tertiary structure of the enzyme. These steric perturbations may consequently impact on the structural integrity and conformational stability of AMY2B.

*GAA* encodes lysosomal acid α-glucosidase, the hydrolase that degrades glycogen within the lysosome (Supplementary Figure 3D-E). The NP_000143.2:p.Arg89His substitution (rs200586324) revealed loss of a hydrogen bond between Arg89 and the backbone oxygen of Val84, contributing to local secondary structure destabilization (Supplementary Figure 3F). Electrostatic potential analysis demonstrated a marked shift from positive to negative potential, potentially affecting protein stability and enzymatic function within the acidic lysosomal environment (Supplementary Figure 3D-F).

*GALE* encodes the UDP-glucose 4-epimerase enzyme, which catalyzes the interconversion of UDP-galactose and UDP-glucose, a critical step in the Leloir pathway of galactose metabolism (Supplementary Figure 3G). Structural analysis of the GALE enzyme in complex with its cofactor NAD⁺ revealed that the wild-type residue Gly158 is situated in close proximity to Tyr157, which acts as the active site proton acceptor (Supplementary Figure 3H). Notably, Tyr157 forms a hydrogen bond with the nicotinamide ring of NAD⁺, highlighting its importance in cofactor binding and catalysis. The NP_000394:p.Gly158Ser (rs374106365) missense mutation, which substitutes the small glycine residue with the larger, polar serine, is predicted to induce significant structural changes. In the mutant structure, the serine residue at position 158 is predicted to form new hydrogen bond interactions with Thr134 and Asn155 (Supplementary Figure 3I). These novel hydrogen bond formations were not observed in the wild-type structure, suggesting that the p.Gly158Ser mutation could potentially alter the local conformational dynamics and active site architecture of GALE.

*GANC* encodes the glucosidase alpha, neutral C enzyme, which plays a crucial role in the lysosomal degradation of glycogen by hydrolyzing the α-1,4 and α-1,6 glycosidic linkages (Supplementary Figure 3J). The NP_937784.2:p.Asn371Tyr (rs751368632) missense mutation in *GANC* is predicted to cause significant steric hindrance within the enzyme's structure. The substitution of the smaller, uncharged asparagine residue at position 371 (Supplementary Figure 3K) with the larger, aromatic tyrosine side chain is anticipated to result in clashes with the neighboring residues Asp398 and Trp370 (Supplementary Figure 3L). This steric hindrance could disrupt the precise packing and folding of the local structural environment, potentially destabilizing the overall tertiary structure of the enzyme.

*PGM1* encodes phosphoglucomutase-1, which interconverts glucose-1-phosphate and glucose-6-phosphate (Supplementary Figure 3M). The nonsense mutation NP_001166289.1:p.Arg503* nonsense mutation (rs397515423), which eliminates 59 C-terminal amino acids including critical phosphate-coordinating residues Arg503, Ser505, Thr507, and Arg515 (Supplementary Figure 3N). This premature termination removes the entire domain 4, disrupting the essential "heart-shaped" catalytic architecture characteristic of the α-D-phosphohexomutase superfamily (Supplementary Figure 3O). The elimination of this domain prevents the coordinated conformational changes required for substrate binding, phosphoryl transfer, and product release, rendering the truncated protein both structurally unstable and catalytically inactive.

*PHKA1* encodes the phosphorylase b kinase regulatory subunit alpha, skeletal muscle isoform, which plays a crucial role in the regulation of glycogen metabolism by activating glycogen phosphorylase through phosphorylation (Supplementary Figure 3P). Structural analysis revealed an intricate hydrogen bond network involving Arg1124, Asn1116, Asn1065, and Glu1112 (Supplementary Figure 3Q). Specifically, Arg1124 forms a hydrogen bond with Asn1116, which in turn participates in a hydrogen bond interaction with Asn1065. Additionally, Asn1065 engages in a hydrogen bond with Glu1112, forming an interconnected network of hydrogen bonds within this region of the protein. The NP_002628.2:p.Arg1124His (rs782546754) missense mutation, which substitutes the positively charged arginine residue with a histidine, is predicted to disrupt the hydrogen bond interaction with Asn1116 (Supplementary Figure 3R). This disruption could potentially influence the stability of the hydrogen bond network involving Asn1116, Asn1065, and Glu1112, leading to structural perturbations within this region of the enzyme.

*SI* encodes the sucrase-isomaltase enzyme, which plays a crucial role in the digestion and absorption of dietary carbohydrates in the intestinal brush border (Supplementary Figure 3S). A natural variant designated NP_001032.2:p.Gly1073Asp (rs121912616) has been identified in the *SI* gene and is associated with impaired carbohydrate digestion and absorption. This missense mutation involves the substitution of the small, non-polar glycine residue at position 1073 (Supplementary Figure 3T) with the larger, negatively charged aspartic acid residue. Structural analysis revealed that the wild-type residue Gly1073 is situated near a loop region containing Ser1088 and Trp1089. The introduction of the bulkier aspartic acid side chain in the p. Gly1073Asp variant is predicted to cause significant steric hindrance with this loop, potentially disrupting the local conformation and stability of the enzyme (Supplementary Figure 3U).


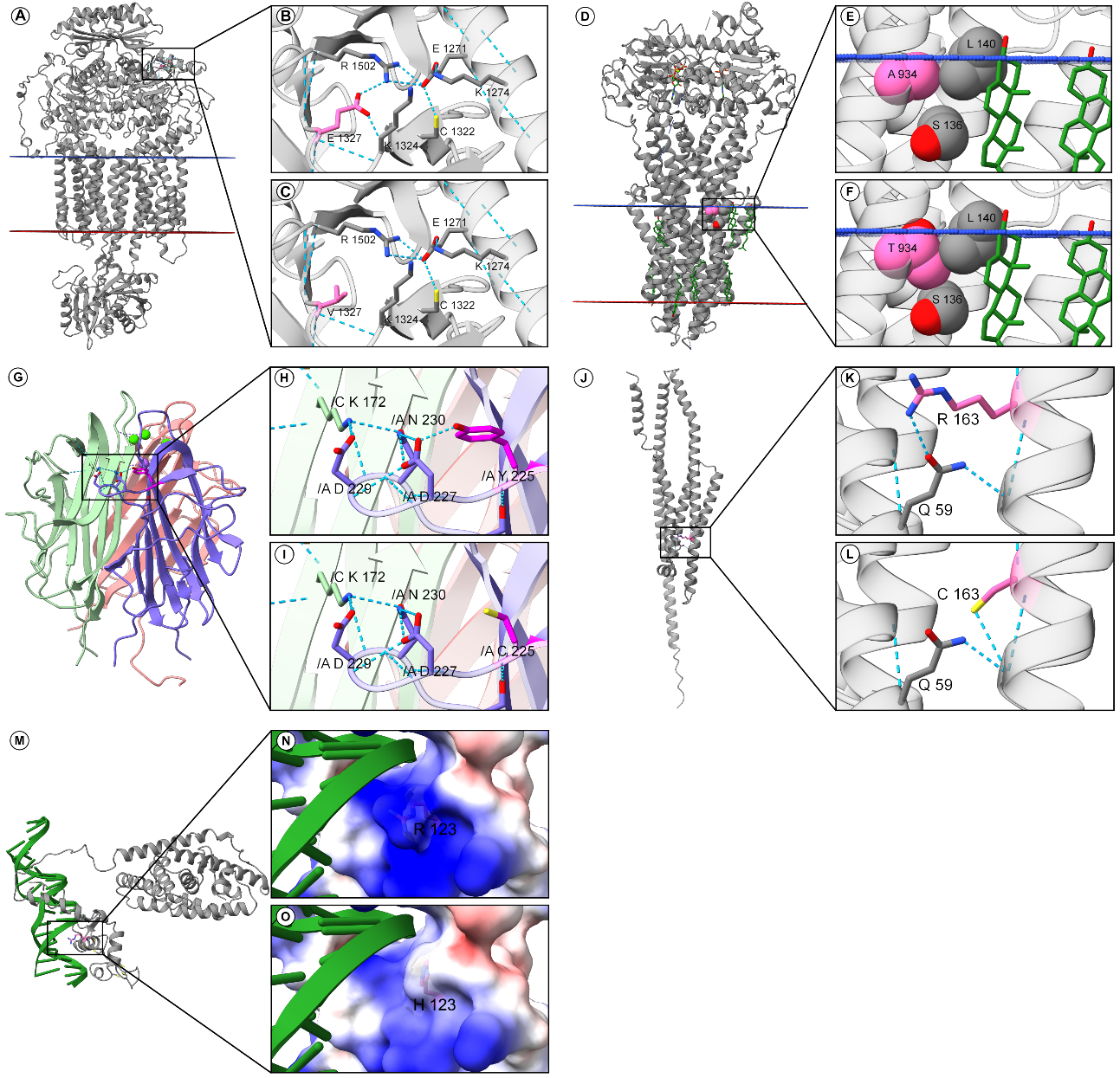


**Supplementary Figure 4. Expanded structural consequences of variants in cholesterol/lipoprotein metabolism genes.**

*In silico* analyses were conducted to model the effects of missense variants on protein conformation and function, focusing on key residues and potential disruptions in active sites or binding pockets:

**(A-C)** Cholesterol transporter ABCA5 and NP_758424.1:p.Glu1327Val: (A) ABCA5 model with predicted membrane orientation. (B) Close-up of Glu1327. (C) Variant model: p.Glu1327Val disrupts a salt bridge with Arg1502 and hydrogen bonds with Lys1324, affecting a network that includes Glu1271, Lys1274, and Cys1322.

**(D-F)** Phospholipid transporter ABCB4 and NP_000434.1:p.Ala934Thr (rs61730509): (D) Electron microscopy structure (E) Close-up of Ala934. (F) Variant model: Ala934Thr may cause steric clashes with Ser136 and Leu140 on an adjacent α-helix.

**(G-I)** Adiponectin (ADIPOQ) and NP_001171271.1:p.Tyr225Cys (rs754228738): (G) Crystal structure of the trimeric globular domain. (H) Close-up of Tyr225, which forms hydrogen bonds with Asp227, Asn230, and Asp229, interacting with Lys172 of an adjacent monomer. (I) Variant model: p.Tyr225Cys may disrupt this hydrogen bond network.

**(J-L)** Cholesterol transporter APOE and NP_000032.1:p.Arg163Cys (rs769455): (J) APOE model. (K) Close-up of Arg163. (L) Variant model: p.Arg163Cys may disrupt a hydrogen bond between Arg163 and Gln59 on adjacent α-helices.

**(M-O)** NR1H3 and NP_005684.2:p.Arg123His (rs774079609): (M) NR1H3 model. (N) Close-up of Arg123 within DNA-binding domain, demonstrating strong positive charge distribution (blue) essential for DNA interaction. (O) Variant model: p. Arg123His reduces electrostatic potential and decreases side chains volume available for DNA contact. The electrostatic potential surfaces utilize the standard color scheme: negative potential in red, positive potential in blue, and neutral regions in white.

*ABCA5* encodes an ATP-binding cassette transporter facilitating cholesterol efflux (Supplementary Figure 4A). The sequence variation (no dbSNP Reference ID) promotes the amino acid change NP_758424.1:p.Glu1327Val in the encoded protein. This amino acid is located within the conserved ATP-binding domain (residues 1290–1533) annotated as IPR003439 in the InterPro database (Supplementary Figure 4A). Structural analysis revealed that Glu1327 forms a crucial salt bridge with Arg1502 and hydrogen bonds with the Lys1324 backbone (Supplementary Figure 4B). Furthermore, Arg1502 engages in a hydrogen bonding network with Glu1271, Lys1274, and Cys1322. Substituting the negatively charged, polar Glu1327 with the nonpolar valine would disrupt this complex network of salt bridges and hydrogen bonds, potentially destabilizing the ATP-binding domain (Supplementary Figure 4C).

*ABCB4* encodes a phospholipid transporter associated with cholestatic liver diseases (Supplementary Figure 4D). The NP_000434.1:p.Ala934Thr (rs61730509) variant is located within an intermembrane α-helical region (Supplementary Figure 4E-F). Structural analysis suggested that the bulkier, polar Thr934 could clash with Ser136 and Leu140 on an adjacent helix, disrupting the precise packing and folding of the intermembrane α-helices (Supplementary Figure 4F). This structural perturbation could adversely affect the integrity of the transmembrane domain, impairing ABCB4's function as an ABC transporter.

*ADIPOQ* encodes the adipokine adiponectin, a regulator of lipid and glucose metabolism. The NP_001171271.1:p.Tyr225Cys (rs754228738) variant is located within the globular head domain of the homotrimeric adiponectin structure (PDB ID: 6U66) (Supplementary Figure 4G). Tyr225 participates in a hydrogen bond network with Asp227, Asn230, and Asp229, which interact with Lys172 of an adjacent monomer (Supplementary Figure 4H). Substituting the bulky, aromatic Tyr225 with the smaller, sulfhydryl-containing cysteine could disrupt this intricate hydrogen bond network, potentially destabilizing the multimeric assembly and perturbing adiponectin's structural integrity (Supplementary Figure 4I).

APOE (Apolipoprotein E) plays a pivotal role in cholesterol homeostasiss (Supplementary Figure 4J). The NP_000032.1:p.Arg163Cys (rs769455) variant is predicted to disrupt a hydrogen bond with Gln59, as Arg163 and Gln59 are situated on adjacent α-helices (Supplementary Figure 4K-L). Notably, Arg163 is part of a critical heparin-binding site (residues 162–165). Introducing the smaller, uncharged cysteine at position 163 could alter APOE's heparin-binding affinity and specificity. Disrupting the hydrogen bond with Gln59 and the potential impact on heparin binding could lead to structural destabilization and impaired functionality of APOE.

*NR1H3* encodes liver X receptor-α, a nuclear receptor that controls lipid-responsive transcription (Supplementary Figure 4M). The variant NP_005684.2:Arg123His (rs774079609), positioned within the DNA-binding domain in direct contact with target DNA sequences, demonstrated compromised binding capacity through reduction of positive electrostatic potential and decreased side chain volume available for DNA interaction (Supplementary Figure 4N-O). This structural alteration is predicted to affect binding affinity and transcriptional regulatory function in lipid homeostasis pathways.
